# Polylactide Degradation Activates Immune Cells by Metabolic Reprogramming

**DOI:** 10.1002/advs.202304632

**Published:** 2023-09-22

**Authors:** Chima V. Maduka, Mohammed Alhaj, Evran Ural, Oluwatosin M. Habeeb, Maxwell M. Kuhnert, Kylie Smith, Ashley V. Makela, Hunter Pope, Shoue Chen, Jeremy M. Hix, Christiane L. Mallett, Seock‐Jin Chung, Maxwell Hakun, Anthony Tundo, Kurt R. Zinn, Kurt D. Hankenson, Stuart B. Goodman, Ramani Narayan, Christopher H. Contag

**Affiliations:** ^1^ Comparative Medicine & Integrative Biology Michigan State University East Lansing MI 48824 USA; ^2^ Department of Biomedical Engineering Michigan State University East Lansing MI 48824 USA; ^3^ Institute for Quantitative Health Science & Engineering Michigan State University East Lansing MI 48824 USA; ^4^ Department of Chemical Engineering & Materials Science Michigan State University East Lansing MI 48824 USA; ^5^ School of Packaging Michigan State University East Lansing MI 48824 USA; ^6^ Department of Orthopedic Surgery University of Michigan Medical School Ann Arbor MI 48109 USA; ^7^ Department of Orthopedic Surgery Stanford University Stanford CA 94063 USA; ^8^ Department of Bioengineering Stanford University Stanford CA 94305 USA; ^9^ Department of Microbiology & Molecular Genetics Michigan State University East Lansing MI 48864 USA

**Keywords:** biocompatibility, immune cells, metabolic reprogramming, polylactide, tissue regeneration

## Abstract

Polylactide (PLA) is the most widely utilized biopolymer in medicine. However, chronic inflammation and excessive fibrosis resulting from its degradation remain significant obstacles to extended clinical use. Immune cell activation has been correlated to the acidity of breakdown products, yet methods to neutralize the pH have not significantly reduced adverse responses. Using a bioenergetic model, delayed cellular changes were observed that are not apparent in the short‐term. Amorphous and semi‐crystalline PLA degradation products, including monomeric l‐lactic acid, mechanistically remodel metabolism in cells leading to a reactive immune microenvironment characterized by elevated proinflammatory cytokines. Selective inhibition of metabolic reprogramming and altered bioenergetics both reduce these undesirable high cytokine levels and stimulate anti‐inflammatory signals. The results present a new biocompatibility paradigm by identifying metabolism as a target for immunomodulation to increase tolerance to biomaterials, ensuring safe clinical application of PLA‐based implants for soft‐ and hard‐tissue regeneration, and advancing nanomedicine and drug delivery.

## Graphical abstract

Polylactide (PLA) is a choice biomaterial for many biomedical applications. Unfortunately, adverse immune responses, whose mechanisms are poorly understood, remain significant limitations to the clinical application of PLA. It is discovered that the metabolism of immune cells surrounding PLA is altered in a manner that propels adverse responses. Thus, targeting metabolic pathways offers novel immunomodulation strategies, enabling the safe application of PLA‐based implants.

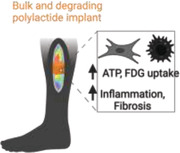

## 1 Introduction

Polylactide (PLA) is the most widely utilized biopolymer,^[^ ^1^ ^]^ with applications in nanotechnology, drug delivery, and adult reconstructive surgery for tissue regeneration. However, after surgical implantation, PLA elicits adverse immune responses in up to 44% of human patients, often requiring further interventions.^[^ ^2^, ^3^ ^]^ In animals, a 66% incidence of excessive fibrosis with capsules from long‐term inflammation which significantly limit implant‐tissue integration has been reported.^[^ ^4^ ^]^ PLA degrades by hydrolysis into d‐ or l‐lactic acid, with semi‐crystalline PLA degrading slower and tending to contain less D‐content than amorphous PLA.^[^ ^1^, ^5^ ^]^ Adverse responses to PLA are exacerbated by mechanical loading and increasing implant size,^[^ ^6^ ^]^ and occur after prolonged exposure to large amounts of PLA degradation products.^[^ ^2^, ^7^, ^8^, ^9^ ^]^ It is speculated that adverse responses are mediated by PLA degradation reducing pH in surrounding tissue,^[^ ^10^ ^]^ the historical basis of which involved *Photobacterium phosphoreum*.^[^ ^11^ ^]^ This bacterium expresses a luciferase whose reduced metabolic activity, measured by bioluminescence, can infer toxicity. In this study, breakdown products (extract) of PLA were obtained either in sterile water or Tris buffer; addition of acidic extract correlated with reduced luminescence. However, the study was not performed on mammalian cells, did not reflect the buffered in vivo microenvironment or simulate prolonged exposure times to accumulated PLA degradation products. Establishing that a decrease in pH correlates with PLA degradation has informed the current strategy in regenerative medicine to neutralize acidic PLA degradation products both in vitro and in vivo using polyphosphazene,^[^ ^12^ ^]^ calcium carbonate, sodium bicarbonate, and calcium hydroxyapatite salts,^[^ ^10^ ^]^ bioglass^[^ ^13^ ^]^ and composites containing alloys or hydroxides of magnesium^[^ ^14^ ^]^ despite reports of failures.^[^ ^15^ ^]^ The lack of a clearly described mechanism of immune cell activation by PLA degradation remains a major obstacle in the safe application of large‐PLA‐based implants in load‐bearing applications as reflected by their paucity in FDA approvals,^[^ ^16^ ^]^ and in soft tissue surgery where neutralizing ceramics cannot be applied.^[^ ^17^ ^]^

Metabolic reprogramming refers to significant changes in oxidative phosphorylation and glycolytic flux patterns and is a driver of fibrosis and bacterial lipopolysaccharide (LPS)‐induced inflammation.^[^ ^18^, ^19^ ^]^ Here we set out to establish a molecular mechanism that directly links metabolic reprogramming to inflammation and fibrosis, consequent to cellular interactions with PLA degradation products. Foremost, we develop and validate a bioenergetic model of prolonged immune cell interaction with accumulated PLA degradation products. Only after prolonged exposure to amorphous or semi‐crystalline PLA degradation products did macrophages and fibroblasts mechanistically undergo metabolic reprogramming and marked bioenergetic changes, with higher PLA crystallinity delaying onset. Using our model, we observed that PLA breakdown products markedly increase proinflammatory cytokine expression in primary macrophages through lactate signaling. Targeting different glycolytic steps using small molecule inhibitors modulated proinflammatory and stimulated anti‐inflammatory cytokine expression by inhibiting metabolic reprogramming and altered bioenergetics in a dose‐dependent manner. This process is highly specific and not cytotoxic to surrounding unaffected immune cells. Further, we demonstrate that the use of the small molecule inhibitors imbedded in PLA implants substantiated our hypothesis of controlling the inflammatory response in vivo. Our findings establish a new biocompatibility paradigm by identifying altered metabolism as a target for immunomodulation of PLA‐based implants, fundamentally differing from previous strategies aimed at neutralizing PLA. Therefore, major advances in the use of PLA for human and veterinary applications are anticipated.

## 2 Results

### 2.1 Bioenergetic Model for Evaluating Cellular Responses to PLA Degradation

**Figure 1:**
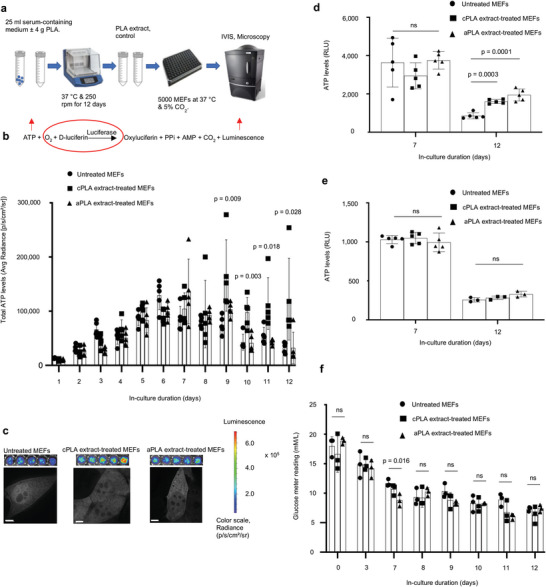
Bioenergetic (ATP) levels are elevated in mouse embryonic fibroblasts (MEFs) only after prolonged exposure to polylactide (PLA) degradation products (extract). a) Workflow showing our in vitro bioenergetic model. b) Keeping luciferase, oxygen, and d‐luciferin levels constant (red circle) allows for changes in ATP (red arrow) to be measured by luminescence (red arrow). Using in vivo imaging system (IVIS) and in comparison to controls, ATP levels in live cells are increased in blasticidin‐eGFP‐luciferase (BGL)‐transfected MEFs after prolonged exposure to crystalline PLA (cPLA) degradation products. c) Representative microscopic (scale bars, 5 µm) and IVIS images show differential nucleoli number and luminescence, respectively. d) Measuring ATP in cell lysates of wild‐type MEFs revealed that prolonged exposure to both amorphous PLA (aPLA) and cPLA results in elevated ATP levels. e) Addition of PLA does not affect the biochemical reaction by which ATP is measured. f) Between groups on the same day, glucose levels are similar in our in vitro bioenergetic model. Not significant (ns), mean (SD), n = 5 (Figure 1b,d and day 7 for 1e) or n = 3 ((f) and day 12 for (e)), one‐way ANOVA followed by Tukey's post‐hoc test; 100 µl of control or PLA extract was used.

In vitro degradation of PLA to examine biocompatibility is well characterized and outlined by the International Standard Organization (ISO 10993–5:2009—Biological evaluation of medical devices). To simulate in vivo buffer conditions, breakdown products of PLA, generally referred to as extracts,^[^ ^20^ ^]^ were generated in serum‐containing DMEM medium and used after 12 days (d) of incubation in a shaker at 37 °C (**Figure** **1**a). This in vitro degradation method was designed to mimic PLA degradation in vivo, with agitation to accelerate PLA degradation relative to static methods.^[^ ^21^ ^]^ Due to the buffering inherent in the serum‐containing DMEM medium, there were no changes in pH over the 12 d extraction period for serum‐containing control medium (pH 8.0), amorphous PLA (pH 8.2) and crystalline PLA (pH 8.2) extracts used on cells. On the other hand, extraction in water for the same duration resulted in pH differences between control (pH 8.2), amorphous PLA (pH 7.5), and crystalline PLA (pH 7.6) extracts.

Together, studies in rodents, dogs, and humans indicate that adverse immune responses occur after accumulation of PLA degradation products over several weeks or months.^[^ ^8^, ^22^, ^23^, ^24^ ^]^ To account for these extended exposure times in our model, we cultured immune cells in PLA extract for 12 d, and this required initiating our cultures with small numbers of cells per well in both control and treatment groups to prevent overgrowth of the cultures. Mouse embryonic fibroblasts (NIH 3T3 cells) were stably transfected with a Sleeping Beauty transposon plasmid (pLuBIG) having a bidirectional promoter driving a modified firefly luciferase gene (fLuc) and a fusion gene encoding a Blasticidin‐resistance marker (BsdR) linked to eGFP (BGL).^[^ ^25^ ^]^ Seeding the same cell numbers across control and treatment groups resulted in constant levels of luciferase and we exposed cells to equal levels of d‐luciferin and oxygen in all assays. In this manner, ATP was rate‐limiting and changes in ATP were measured by bioluminescence using in vivo imaging system (IVIS; Figure 1b). The use of bioluminescence as an indicator of ATP levels was inexpensive, rapid (on the order of seconds), and allowed for high throughput temporal bioenergetic analysis in live cells. Additionally, in our model, each well of a 96‐well plate had a total of 200 µl of medium, of which 100 µl was freshly prepared. The additional 100 µl for control wells was medium that had been in the shaker at 37 °C for 12 d to account for potential nutrient degradation that could confound results. Similarly, the additional 100 µl for treatment wells was a medium in which PLA had been degraded under the same conditions. Dose‐bioenergetic response of amorphous and crystalline PLA extracts revealed altered ATP levels for all tested doses (Figure S1a, Supporting Information). Therefore, we selected 100 or 150 µl of extract, as indicated in figure legends, to mimic the accumulation of voluminous PLA breakdown products.^[^ ^2^, ^7^ ^]^

Highly crystalline and amorphous PLA samples were selected for their high molecular weights and represent a range of physicochemical properties (crystallinity, stereochemistry, degradation period) which constitute important considerations in selecting PLA for hard and soft tissue engineering.^[^ ^8^, ^10^, ^24^ ^]^ Before using these PLA materials, we authenticated their physicochemical and thermal properties (Table S1, Supporting Information). Lastly, we used the non‐transformed, immortalized NIH 3T3 fibroblast cell line that typifies primary fibroblasts, as well as primary bone‐marrow‐derived macrophages, both of which are key cellular mediators of prolonged inflammation and excessive fibrosis that occur in response to PLA degradation.^[^ ^12^, ^22^ ^]^

### 2.2 Bioenergetics is Altered in Immune Cells after Exposure to PLA Degradation Products

Unlike in the short‐term (days 0–5), prolonged (days 6–12) exposure of fibroblasts to either amorphous or crystalline PLA increased ATP levels in live cells (Figure 1b,c). Upon high‐resolution z‐stack imaging, there were apparent changes in nucleoli number (Figure 1c) after prolonged exposure to either amorphous or crystalline PLA extract, which could represent a stress response.^[^ ^26^ ^]^ To exclude the possibility that changing luciferase expression (by transcription or translation) was responsible for observed bioenergetic changes, we lysed wild‐type cells after exposure to PLA extract and added controlled amounts of luciferase and d‐luciferin in the standard ATP assay. Moreover, measuring ATP levels in live cells by IVIS is constrained by parameters inherent to live cells. Lysed cells allow for the measurement of ATP from all organellar compartments, and are not constrained by d‐luciferin uptake, revealing more information than measurements in live cells. By day 12, there was a 1.9‐ and 2.3‐fold increase in ATP levels among cells exposed to crystalline and amorphous PLA extract, respectively (Figure 1d). To exclude the possibility that PLA extracts affect the biochemical reaction (Figure 1b) underlying bioenergetic measurements, fibroblasts were cultured for different time points. Thereafter, lysed fibroblasts were exposed to d‐luciferin, luciferase, and control or PLA extract at the same time. No difference in ATP levels was observed, confirming that treatment with PLA extract did not affect this biochemical reaction (Figure 1e).

Declining ATP levels from day 0 to 12 are likely due to changing glucose levels.^[^ ^27^ ^]^ To determine whether glucose levels changed between groups on the same day because of the extended exposure times in our model, glucose meter readings were optimized in a mammalian cell culture medium (Figure S1b, Supporting Information). Glucose levels were similar between groups on each day (Figure 1f). On day 7, when untreated groups had higher glucose levels (Figure 1f), corresponding bioenergetic measurement revealed that PLA extract‐treated fibroblasts had higher ATP levels (Figure S1c, Supporting Information), excluding changing glucose levels as a confounding factor in our bioenergetic model. Because NIH 3T3 cells are normal immortalized fibroblasts, changing cell number from proliferation could account for bioenergetic changes. To exclude this, we optimized the crystal violet assay for cell number measurement^[^ ^28^ ^]^ in fibroblasts (Figure S2a, Supporting Information). Next, we isolated mouse primary bone marrow‐derived macrophages (BMDMs) which, unlike NIH 3T3 cells, do not proliferate.^[^ ^29^ ^]^

**Figure 2:**
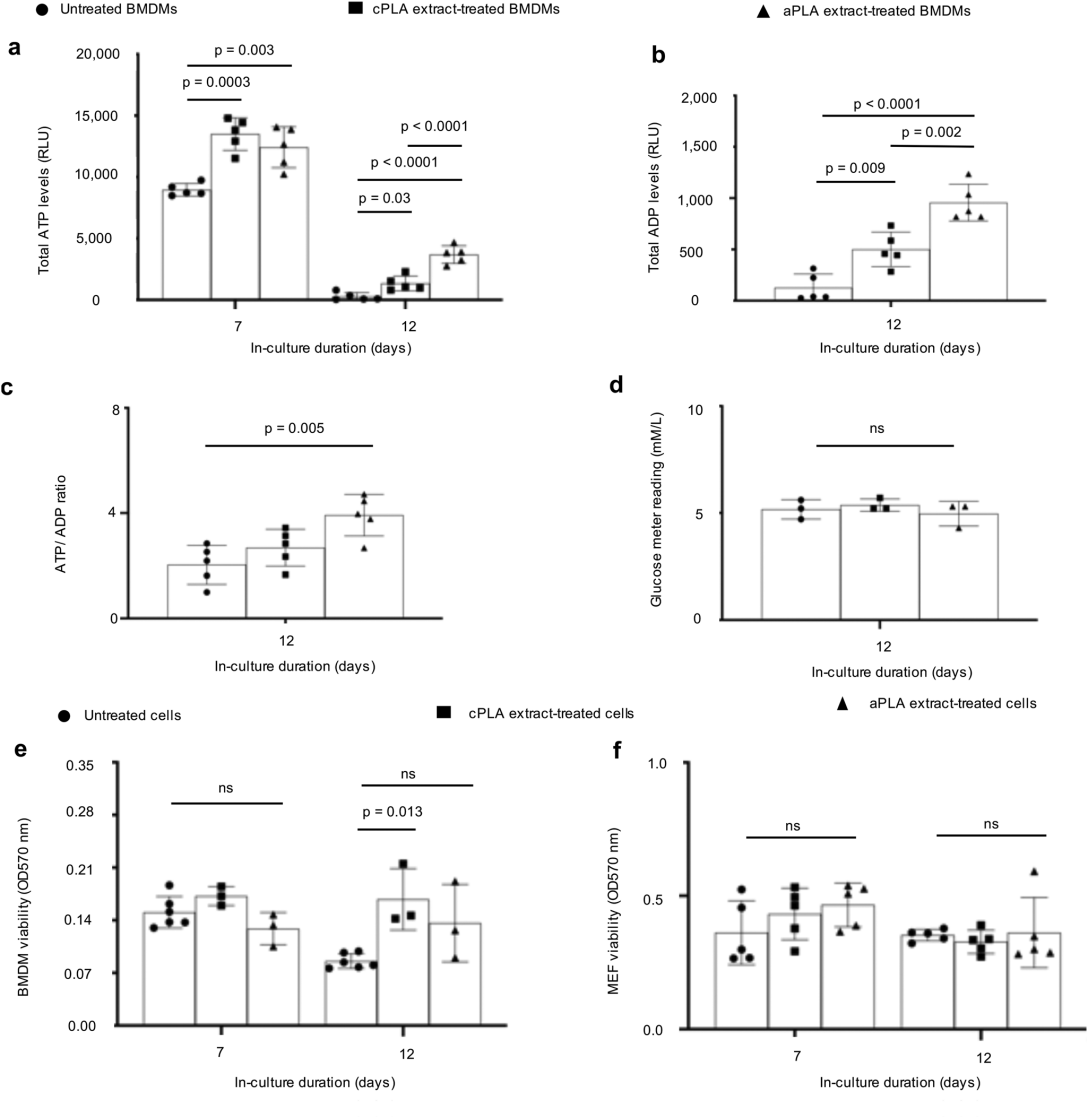
Bioenergetics is increased in primary bone marrow‐derived macrophages (BMDMs) after prolonged exposure to polylactide (PLA) degradation products (extract). a) ATP levels b) ADP levels, c) and ATP/ADP ratios are increased in BMDMs after prolonged exposure to amorphous PLA (aPLA) or crystalline PLA (cPLA) degradation products (extracts) in comparison to controls. d) Glucose levels between groups on day 12 are similar. e,f) Cell numbers between groups are similar for BMDMs (e) and MEFs (f). Not significant (ns), mean (SD), n = 5 (a–c,f), n = 3, (d), n = 3–6 (e), one‐way ANOVA followed by Tukey's post‐hoc test; 100 µl of control or PLA extract was used.

Both ATP^[^ ^30^ ^]^ and ADP^[^ ^31^ ^]^ metabolism and ratios are crucial in inflammatory conditions. In BMDMs and consistent with our observations in fibroblasts, we observed marked increases in ATP and ADP levels (**Figure** **2**a,b) or ATP/ADP ratios (Figure 2c) which were not due to changing glucose levels (Figure 2d). After optimizing the crystal violet assay for macrophages (Figure S2b, Supporting Information), overall, cell numbers could not account for observed bioenergetic changes (Figure 2e). Furthermore, fibroblast numbers were similar for cultures that were untreated or exposed to PLA extracts (Figure 2f), excluding changing cell numbers as a confounder in our model.

### 2.3 Exposure of Macrophages to PLA Breakdown Products Selectively Results in Metabolic Reprogramming

**Figure 3:**
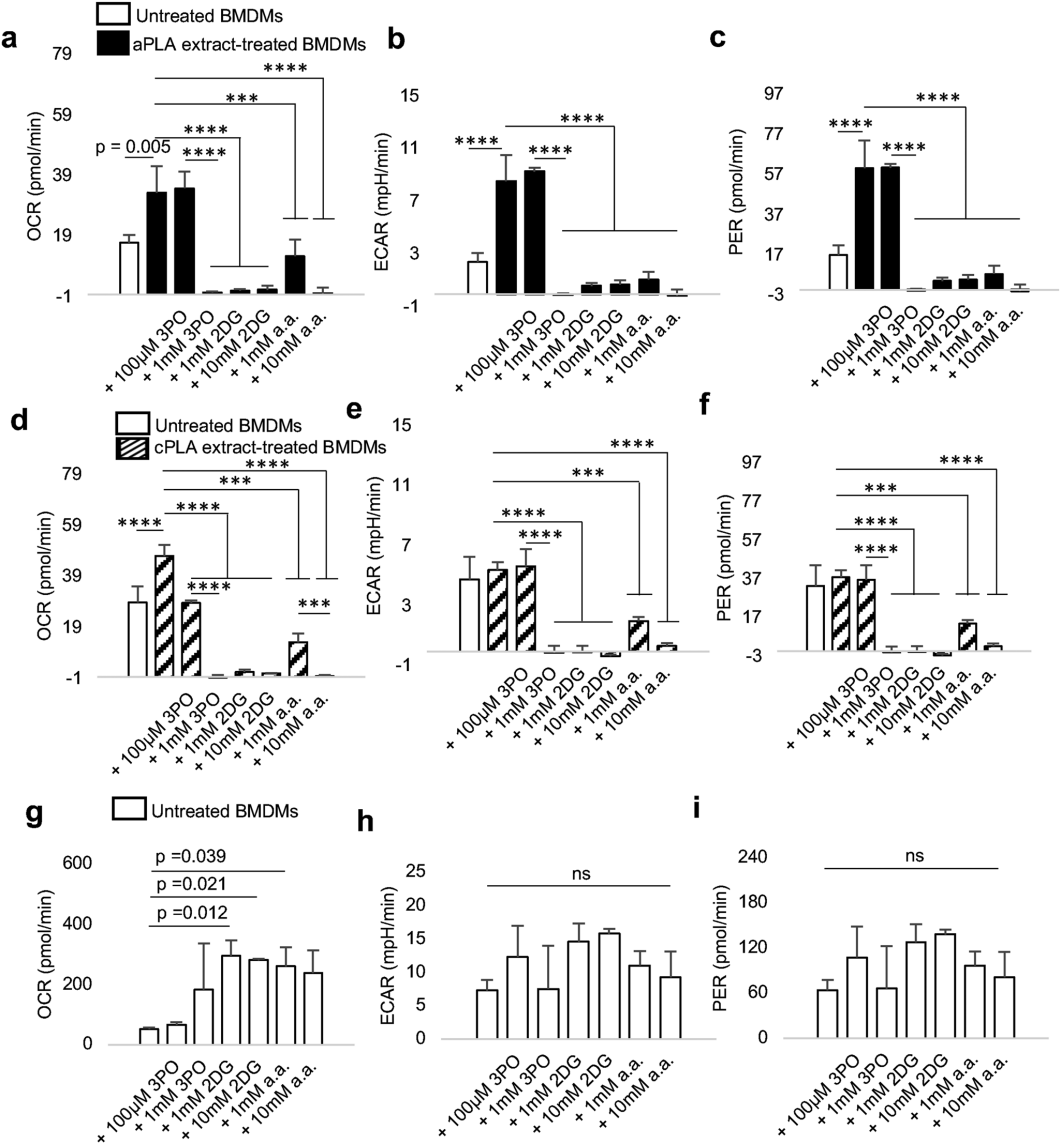
Functional metabolic indices are altered in primary bone marrow‐derived macrophages (BMDMs) after prolonged exposure to polylactide (PLA) degradation products (extract), and can be modulated by glycolytic inhibitors. a–c) Following exposure to amorphous PLA (aPLA) extract, oxygen consumption rate (OCR) (a) extracellular acidification rate (ECAR) (b) and proton efflux rate (PER) (c) are increased relative to controls, and this abnormal increase can be dose‐dependently controlled by various small molecule inhibitors. d–f) OCR (d) and not ECAR (e) and PER (f) are increased relative to controls in groups exposed to crystalline PLA (cPLA) extract, and functional metabolic indices can be controlled by pharmacologic inhibitors of glycolysis. g) Compensatory increase in OCR occurs in untreated BMDMs after treatment with some inhibitors. h,i) ECAR (h) and PER (i) are not affected by glycolytic inhibitors in untreated BMDMs. Not significant (ns), ^***^ *p* < 0.001, ^****^ *p* < 0.0001, mean (SD), n = 3, one‐way ANOVA followed by Tukey's post‐hoc test; 3‐(3‐pyridinyl)−1‐(4‐pyridinyl)−2‐propen‐1‐ one (3PO), 2‐deoxyglucose (2DG) and aminooxyacetic acid (a.a.); 100 µl of control or PLA extract was used for 7 days.

To determine the metabolic pathways responsible for the bioenergetic changes we had observed, Seahorse assays were used to measure oxygen consumption rate (OCR), extracellular acidification rate (ECAR), and lactate‐linked proton efflux rate (PER) in a customized medium (pH 7.4); this technique has not been previously used to examine PLA‐induced adverse responses. PLA extract was removed and washed off the cells prior to running the Seahorse assay at a pH 7.4. Seahorse assays measure ECAR as an index of glycolytic flux, OCR as an index of oxidative phosphorylation, and PER as an index of monocarboxylate transporter function^[^ ^32^ ^]^ in live cells; and are used to assess for metabolic reprogramming.^[^ ^33^, ^34^, ^35^ ^]^ Primary BMDMs exposed to amorphous PLA extract were metabolically altered, showing a two‐fold increase in oxidative phosphorylation (OCR; **Figure** **3**a), 3.5‐fold increase in glycolytic flux (ECAR; Figure 3b), and 3.5‐fold increase in monocarboxylate transporter activity (PER; Figure 3c) in comparison to untreated BMDMs. Similar amounts (100 µl) of crystalline PLA extract resulted in a 1.6‐fold increase in OCR (Figure 3d) but no change in ECAR (Figure 3e) or PER (Figure 3f). However, higher amounts (150 µl) of crystalline PLA extract resulted in 3.2‐, 3.8‐, and 3.8‐fold increases in OCR, ECAR, and PER, respectively (Figure S3a–c, Supporting Information) compared to controls, suggesting that greater volume of PLA extract is required for reprogramming using crystalline than amorphous PLA.

Next, we targeted different steps in the glycolytic pathway using three small molecule inhibitors: 3‐(3‐pyridinyl)−1‐(4‐pyridinyl)−2‐propen‐1‐one (3PO), 2‐deoxyglucose (2DG) and aminooxyacetic acid (a.a.). Whereas 3PO specifically inhibits 6‐ phosphofructo‐2‐kinase which is the rate‐limiting glycolytic enzyme,^[^ ^36^ ^]^ 2DG inhibits hexokinase, the first enzyme in glycolysis,^[^ ^35^ ^]^ and aminooxyacetic acid prevents uptake of glycolytic substrates.^[^ ^37^ ^]^ In a dose‐dependent manner, 3PO, 2DG, and a.a. inhibited metabolic reprogramming following exposure to amorphous PLA (Figure 3a–c) or crystalline PLA extract (Figure 3f), but not in untreated BMDMs (Figure 3g–i). This demonstrates cellular uptake of 3PO, 2DG, and a.a., yet with selective pharmacologic effects. Notably and under the same experimental conditions, cell viability was not reduced in untreated BMDMs after exposure to glycolytic inhibitors (Figure S2c, Supporting Information), demonstrating the absence of cytotoxicity.^[^ ^28^ ^]^ However, when BMDMs were treated with amorphous or crystalline PLA extract, where metabolism was abnormally remodeled, 3PO, 2DG, and a.a. mildly, but selectively, reduced cell viability (Figure S2d, Supporting Information). Therefore, pharmacologically targeting altered metabolism in primary BMDMs following exposure to PLA extract is highly specific with limited toxicity to immune cells that have normal metabolic profiles.

### 2.4 Fibroblasts are Glycolytically Reprogrammed after Exposure to PLA Breakdown Products

**Figure 4:**
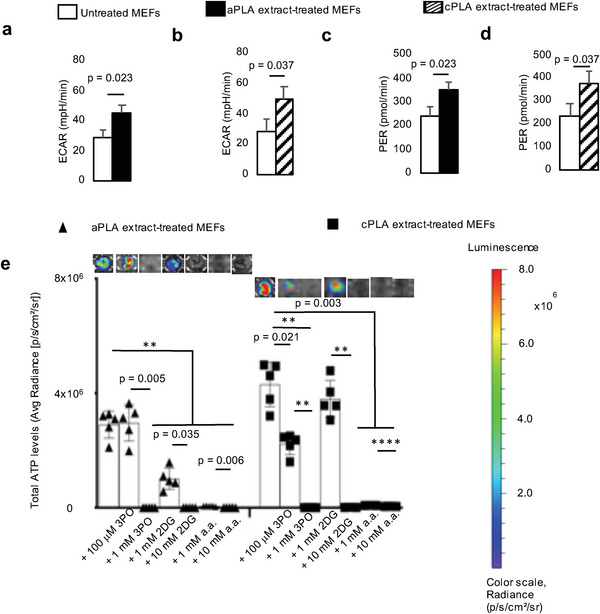
Functional metabolism is altered in mouse embryonic fibroblasts (MEFs) after exposure to polylactide (PLA) degradation products (extract). a,b) Following exposure to amorphous PLA (aPLA; a) or crystalline PLA (cPLA; b) extracts, extracellular acidification rate (ECAR) is increased. c,d) Proton efflux rate (PER) is elevated in MEFs after exposure to aPLA (c) or cPLA (d) extract. e) Bioenergetic levels in MEFs exposed to aPLA or cPLA extracts are decreased in a dose‐dependent manner by 3‐(3‐pyridinyl)−1‐(4‐pyridinyl)−2‐propen‐1‐one (3PO), 2‐deoxyglucose (2DG) and aminooxyacetic acid (a.a.; representative wells are shown). ^**^ *p* = 0.002, ^****^ *p* < 0.0001, mean (SD), n = 3 (a–d), n = 5 (e), two‐tailed unpaired *t*‐test or Brown–Forsythe and Welch ANOVA followed by Dunnett's T3 multiple comparisons test; 100 µl of control or PLA extract was used for 7 days.

After prolonged exposure of fibroblasts to amorphous and crystalline PLA extracts, glycolytic flux (ECAR; **Figure** **4**a,b) is increased by 1.6‐ and 1.7‐fold, respectively. Furthermore, monocarboxylate transporter function is increased in amorphous or crystalline PLA extract‐treated fibroblasts by 1.6‐ and 1.5‐fold, respectively (Figure 4c,d). However, oxidative phosphorylation remains similar between untreated fibroblasts and cells exposed to amorphous or crystalline PLA extracts (OCR; Figure S4a,b, Supporting Information). Remarkably, increased bioenergetic (ATP) levels in amorphous or crystalline PLA extract‐treated fibroblasts are inhibited by 3PO, 2DG, and a.a. in a temporal and dose‐dependent manner (Figure 4e; Figure S4c, Supporting Information).

### 2.5 Short‐ and Long‐Term Exposure to l‐Lactic Acid Alters Bioenergetics and Results in Metabolic Reprogramming

As previously reported for short‐term hydrolytic degradation of PLA,^[^ ^8^ ^]^ there was no reduction in the mass of PLA after our 12 d extraction, but there were detectable changes in molecular weight (Table S2, Supporting Information). Using the standard d/l‐lactic acid enzyme‐based determination assays could not effectively measure levels in a serum‐containing medium. However, in milliQ water and relative to controls, we observed a 7.8‐ and 5.2‐fold increase in l‐lactic acid in amorphous and crystalline PLA extracts, respectively, although these increments were not significant (Table S3 and Figure S5a,b, Supporting Information). These data followed the trend observed using liquid chromatography‐electrospray ionization mass spectrometry, where amorphous PLA tended to degrade faster than crystalline PLA, and where we observed various oligomers of lactic acid present in extracts derived from milliQ water (Figure S5c, Supporting Information). Additionally, we observed a 2.7‐ and 2.8‐fold increase in d‐lactic acid in amorphous and crystalline PLA extracts, respectively (Table S3, Supporting Information). Therefore, we exposed BMDMs to various doses of l‐lactic acid, ranging from 2.5‐ to 15‐fold higher levels in comparison to untreated cells. In addition, we measured corresponding pH levels: Untreated medium (pH 8.01), 2.5 mm (pH 7.47), 5 mm (pH 7.19), 10 mm (pH 6.84), and 15 mm (pH 6.65) l‐lactic acid‐containing DMEM medium.

**Figure 5:**
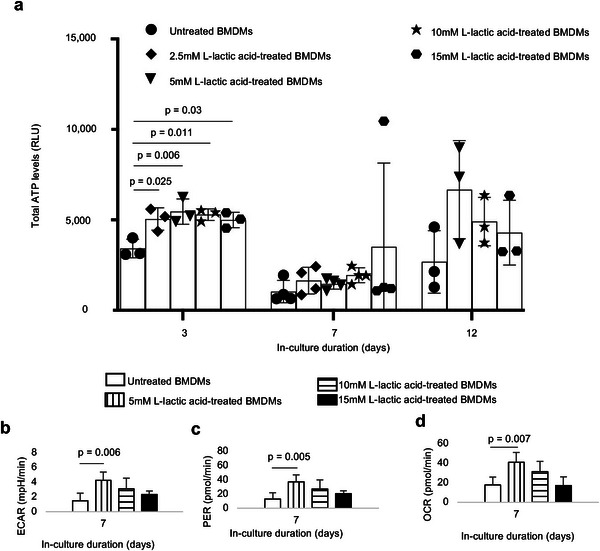
Treatment of primary bone marrow‐derived macrophages (BMDMs) with l‐lactic acid altered bioenergetic (ATP) levels and functional metabolism. a) Treatment with different doses of monomeric Llactic acid resulted in changes in ATP levels. b–d) Following exposure to l‐lactic acid extracellular acidification rate (ECAR, b), proton efflux rate (PER, c) and oxygen consumption rate (OCR, d) are increased. One‐way ANOVA followed by Tukey's post‐hoc test, mean (SD), n = 3–4 (a), n = 5 (b–d).

We observed that bioenergetic levels are altered in the short‐term (day 3; **Figure** **5**a) for all doses of l‐lactic acid treatment, resulting in a 1.5 to 1.6‐fold increase in ATP levels. After prolonged (day 7) exposure to l‐lactic acid and even when bioenergetic alterations were not apparent, glycolytic flux (ECAR; Figure 5b), monocarboxylate transporter function (PER; Figure 5c) and oxidative phosphorylation (OCR; Figure 5d) were increased by 2.8‐, 2.8‐, and 2.3‐fold, mechanistically reproducing observations made with amorphous and crystalline PLA extracts in our bioenergetic model. Moreover, these changes were not dependent on alterations in cell number (Figure S5d, Supporting Information). Of note, highly acidic groups (10–15 mm l‐lactic acid) did not result in a reduction in viability of primary macrophages either at day 7 or 12, relative to controls (Figure S5d, Supporting Information).

### 2.6 Glycolytic Inhibition Modulates Proinflammatory and Stimulates Anti‐Inflammatory Cytokine Expression

**Figure 6:**
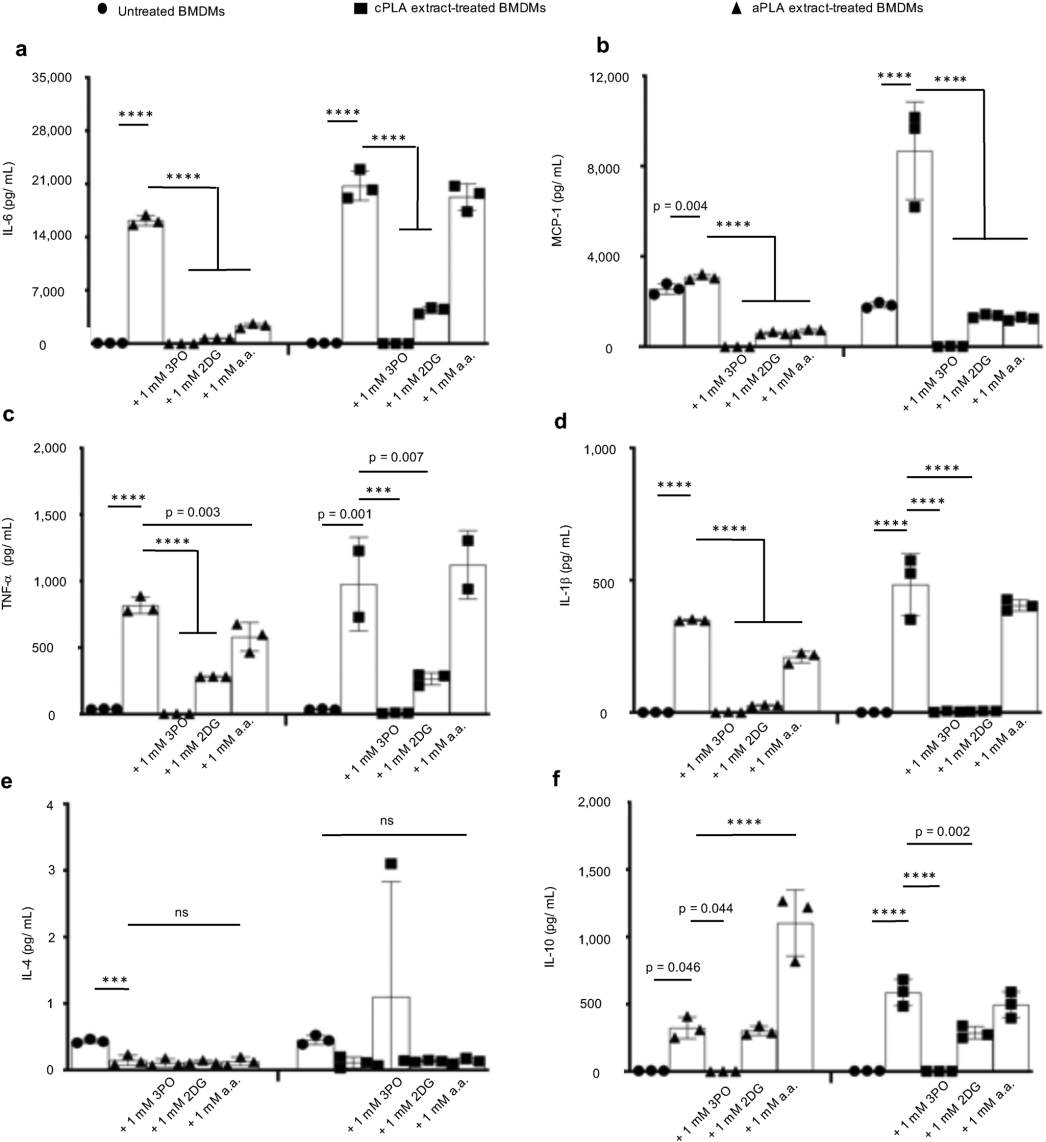
In macrophages exposed to PLA breakdown products, glycolytic inhibitors modulate elevated proinflammatory cytokine expression and stimulate or do not reduce anti‐inflammatory cytokine levels. a–d) Following exposure to amorphous PLA (aPLA) or crystalline PLA (cPLA) extract, primary bone marrow‐derived macrophages (BMDMs) express elevated levels of IL‐6 (a), MCP‐1 (b), TNF‐α (c), and IL‐1β (d) in comparison to untreated BMDMs, and these elevated proinflammatory cytokine levels can be modulated by various small molecule inhibitors of glycolysis. e) Addition of glycolytic inhibitors to PLA does not reduce IL‐4 expression. f) Expression of IL‐10 is increased by inhibiting glycolysis using aminooxyacetic acid (a.a.) in amorphous PLA. Not significant (ns), ^***^ *p* < 0.001, ^****^ *p* < 0.0001, mean (SD), n = 3 in all except the cPLA group in TNF‐α (Figure 6c) where n = 2–3, one‐way ANOVA followed by Tukey's post‐hoc test; 3‐(3‐pyridinyl)−1‐(4‐pyridinyl)−2‐propen‐1‐one (3PO), 2‐deoxyglucose (2DG); 100 µl of aPLA or 150 µl of cPLA extract with corresponding controls were used on day 7.

To determine whether glycolytic inhibition affects proinflammatory (IL‐6, MCP‐1, TNF‐α, IL‐1β and IFN‐γ) and anti‐inflammatory (IL‐4, IL‐10, and 1L‐13) protein expression, we used a magnetic bead‐based chemokine and cytokine assay.^[^ ^38^ ^]^ We observed that prolonged exposure of primary macrophages to amorphous and crystalline PLA extracts resulted in 228‐ and 319‐fold increases, respectively, in IL‐6 protein expression (**Figure** **6**a) compared to untreated macrophages. We confirmed this observation by ELISA (Figure S6a, Supporting Information). Similarly, exposure of macrophages to lactic acid resulted in elevated IL‐6 protein expression by 2.3‐fold (Figure S6a, Supporting Information). Amorphous PLA extracts increased MCP‐1 (Figure 6b), TNF‐α (Figure 6c) and IL‐1β (Figure 6d) levels by 1.2‐, 21‐, and 567‐fold, respectively. Likewise, crystalline PLA extracts increased MCP‐1 (Figure 6b), TNF‐α (Figure 6c), and IL‐1β (Fig 6d) levels by 4.7‐, 27‐, and 1378‐fold, respectively. Abnormally increased levels of IL‐6, MCP‐1, TNF‐α, and IL‐1β were modulated by addition of 3PO, 2DG, or a.a. (Figure 6a–d). Increased MCP‐1 levels in macrophages also occurred after exposure to l‐lactic acid (Figure S6b, Supporting Information). Levels of IFN‐γ and IL‐13 were unchanged by PLA extract (data not shown) but exposure to amorphous PLA extract decreased IL‐4 protein levels by threefold (Figure 6e) relative to untreated macrophages. Remarkably, with the exception of 3PO, IL‐10 expression was either unchanged (crystalline PLA) or increased by 3.4‐fold (amorphous PLA) upon the addition of a.a. (Figure 6f) relative to macrophages exposed to PLA extract.

### 2.7 Increased Radiolabeled Glucose Uptake Occurs in the PLA Microenvironment and Drives Inflammation, In Vivo

**Figure 7:**
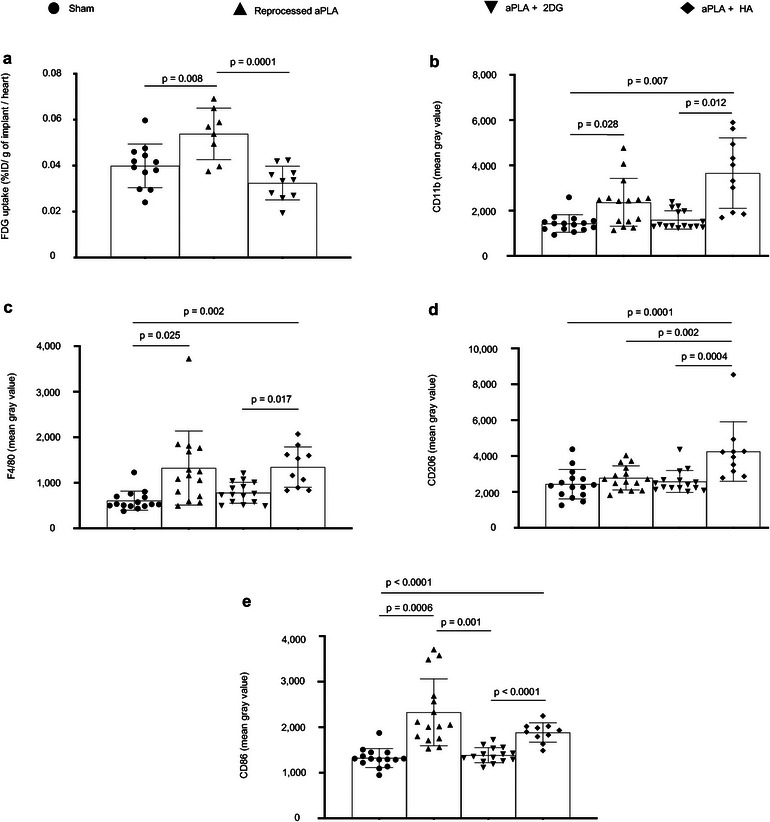
Increased radiolabeled glucose uptake occurs in the polylactide (PLA) microenvironment and drives inflammation in vivo. a) When normalized to heart values, percent injected dose per gram (%ID g^−1^) of biopsied tissues surrounding amorphous PLA (aPLA) implants show higher F‐18 fluorodeoxyglucose (FDG) uptake compared to sham controls; increased FDG uptake is reduced by incorporation of 2‐deoxyglucose (2DG). b,c) Compared to sham controls, mean fluorescence intensity of CD11b (b) or F4/80 (c) is increased following surgical implantation of aPLA or a combination of aPLA and hydroxyapatite (HA), but not a combination of aPLA and 2DG. d) Compared to other groups, CD206 mean fluorescence intensity is increased in aPLA + HA. e) Compared to sham controls, CD86 mean fluorescence intensity is increased following implantation of aPLA; elevated CD86 is decreased by incorporating 2DG but not HA. Mean (SD); Figure 1a, sham (n = 12), aPLA (n = 8), aPLA + 2DG (n = 10); Figure 1b–e, sham (n = 15), aPLA (n = 15), aPLA + 2DG (n = 15), aPLA + HA (n = 10); one‐way ANOVA followed by Tukey's post‐hoc test or Brown–Forsythe and Welch ANOVA followed by Dunnett's T3 multiple comparison test; refer to Experimental Section (in vivo studies, tissue processing and analyses) for more information on n.

Taken together, our in vitro data suggest that metabolic changes drive inflammation arising from PLA degradation. However, in vitro methods for characterizing PLA degradation may not fully simulate the complexity of events in the body. Therefore, we sought to test our hypothesis that metabolic changes drive inflammation in vivo and to test the local efficacy of small molecule metabolic inhibitors. We incorporated 2DG into amorphous PLA (aPLA) by melt‐blending at 190 °C and compared it to aPLA which had been subjected to similar melt‐blending conditions (called reprocessed aPLA). Following melt‐blending, extruded (sterile) filaments (1.75 mm diameter, 1 mm long) were subcutaneously implanted on the back (dorsum) of mice. Sham controls underwent similar surgical exposures but were not implanted with any materials. After 6 weeks, mice were injected with F‐18 fluorodeoxyglucose (FDG) and euthanized; using FDG allows for the evaluation of metabolic reprogramming and inflammation.^[^ ^39^ ^]^ Thereafter, circular biopsies (12 mm in diameter) of full‐thickness skin containing implants were assayed for radioactivity using a gamma counter. Compared to sham controls, skin containing reprocessed aPLA implants had 1.35‐fold increase in FDG uptake, which was abolished in skin containing aPLA+2DG implants (**Figure** **7**a).

Next, we sought to determine the effect of glycolytic inhibition on the recruitment and activation states of macrophages and fibroblasts. To compare the effects of glycolytic inhibition on neutralization techniques, we included a group where hydroxyapatite (HA) was incorporated in aPLA.^[^ ^10^, ^40^ ^]^ Hematoxytin and eosin staining revealed the presence of inflammatory infiltrates in the implant microenvironment (reprocessed aPLA, aPLA+2DG, aPLA+HA) compared to sham controls, suggesting persistent inflammation (Figure S7, Supporting Information).

Chronic inflammation of PLA is principally driven by recruited macrophages.^[^ ^12^, ^22^ ^]^ Therefore, we stained for CD11b and F4/80, established macrophage markers. Compared to sham controls, aPLA resulted in a 1.7‐ and 2.2‐fold increase in CD11b and F4/80 intensities, respectively (Figure 7b,c; Figure S8, Supporting Information). Unlike aPLA+2DG, aPLA+HA increased CD11b and F4/80 intensities by 2.6‐ and 2.2‐fold, respectively, when compared to sham controls (Figure 7b,c). Of note, there was no significant difference in CD11b and F4/80 intensities between aPLA and aPLA+2DG (Figure 7b,c), suggesting similar levels of macrophage recruitment. Furthermore, aPLA+2DG revealed 2.3‐ and 1.7‐fold less CD11b and F4/80 intensities, respectively, compared to aPLA+HA (Figure 7b,c). To determine the activation states of recruited macrophages, we stained for CD206 and CD86, anti‐inflammatory and proinflammatory macrophage markers, respectively.^[^ ^41^ ^]^ Relative to other groups, only aPLA+HA increased CD206 intensity (Figure 7d; Figure S8, Supporting Information), consistent with the bioactivity of HA.^[^ ^42^ ^]^ We observed a 1.8‐fold increase in CD86 intensity with reprocessed aPLA compared to sham controls, consistent with the proinflammatory effects of aPLA (Figure 7e; Figure S8, Supporting Information). Compared to reprocessed aPLA, aPLA+2DG, and not aPLA+HA decreased CD86 intensity (Figure 7e). In fact, there was a 1.4‐fold decrease in CD86 intensity in aPLA+2DG compared to aPLA+HA (Figure 7e).

**Figure 8:**
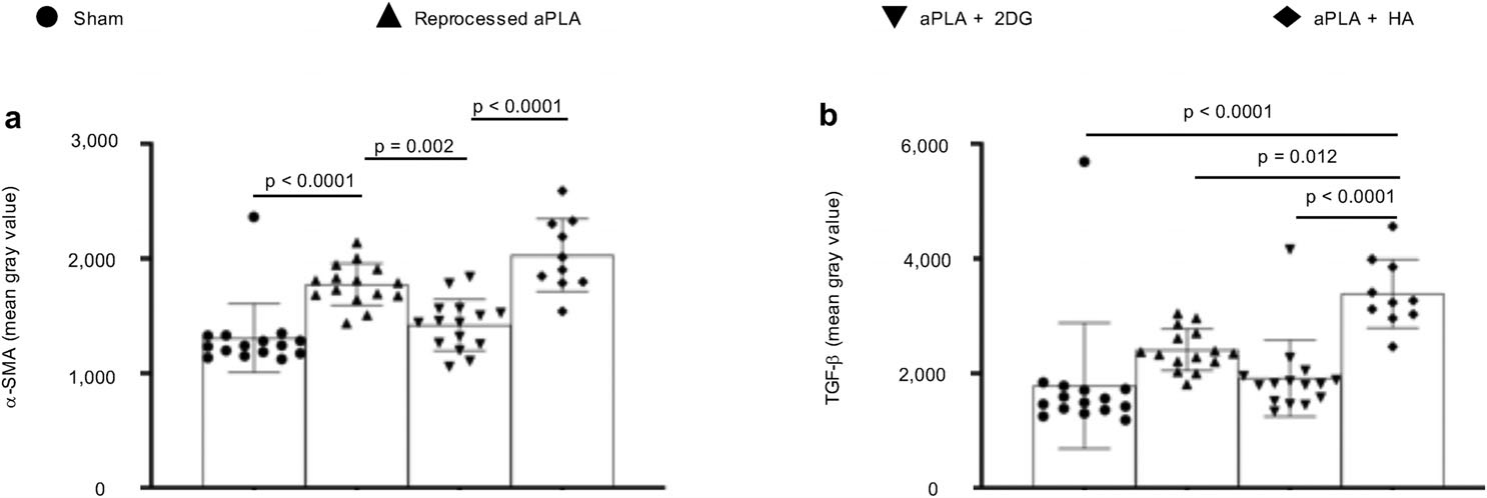
Activation of fibroblasts in the polylactide (PLA) microenvironment is regulated by immunometabolism. a) Compared to sham controls, mean fluorescence intensity of alpha‐smooth muscle actin (α‐SMA) is increased following surgical implantation of amorphous PLA (aPLA) or a combination of aPLA and hydroxyapatite (HA), but not a combination of aPLA and 2‐deoxyglucose (2DG). b) Compared to other groups, mean fluorescence intensity of transforming growth factor‐beta (TGF‐β) is increased in aPLA + HA. Mean (SD); sham (n = 15), aPLA (n = 15), aPLA + 2DG (n = 15), aPLA + HA (n = 10); one‐way ANOVA followed by Tukey's post‐hoc test; refer to Experimental Section (in vivo studies, tissue processing and analyses) for more information on n.

Fibroblasts are a key cellular player of excessive fibrosis around PLA implants,^[^ ^12^, ^22^ ^]^ and their activation in myofibroblast phenotype is marked by α‐SMA and TGF‐β expression.^[^ ^43^ ^]^ We observed a 1.4‐fold increase in α‐SMA intensity with reprocessed PLA compared to sham controls, which was decreased in the aPLA+2DG, but not aPLA+HA group (**Figure** **8**a; Figure S9, Supporting Information). With TGF‐β intensity, aPLA+HA was elevated relative to other groups (Figure 8b; Figure S9, Supporting Information). Compared to aPLA+HA, aPLA+2DG revealed 1.4‐ and 1.8‐fold decrease in α‐SMA and TGF‐β intensities, respectively (Figure 8b).

## 3 Discussion

We describe a bioenergetic model of immune cell activation to PLA degradation, revealing that altered bioenergetics and metabolic reprogramming underlie adverse responses, including persistent inflammation and excessive fibrosis, to PLA breakdown products. For decades, the hypothesis in regenerative medicine has been that acidity drives immune cell activation to PLA degradation.^[^ ^10^ ^]^ However, this observation was founded on correlation and not causation.^[^ ^11^, ^20^ ^]^ Consequently, methods based on neutralizing acidity have been inadequate in controlling adverse responses to PLA degradation.^[^ ^7^, ^15^ ^]^

Importantly, our in vitro model extends the short time periods that have been previously studied.^[^ ^44^ ^]^ By adapting our bioenergetic model for high throughput analysis, we observed delayed immune cell changes not apparent in the short‐term. In patients, PLA slowly degrades into oligomers and monomers of lactic acid. Ultimately, due to bulk degradation, PLA breakdown exceeds immune cellular clearance, resulting in accumulation of oligomers and monomers of lactic acid.^[^ ^24^ ^]^ We illustrate that only after prolonged exposure to PLA degradation products do fibroblasts and macrophages become activated. Mechanistically, PLA degradation not only alters bioenergetic homeostasis in immune cells, but it also results in metabolic reprogramming. We identified PLA degradation products to include monomeric l‐lactic acid and reproduced bioenergetic alterations and metabolic reprogramming using monomeric l‐lactic acid. In addition to monomeric lactic acid, we observed a spectrum of oligomeric lactic acid which appeared more abundant in amorphous than crystalline PLA breakdown products, consistent with faster degradation in crystalline than amorphous PLA biomaterials.^[^ ^6^ ^]^ These additional (oligomeric) breakdown products could account for the difference in fold change produced by monomeric l‐lactic acid versus overall PLA degradation products, suggesting that monomeric lactic acid alone may not fully induce effects elicited by PLA breakdown products.

Following prolonged exposure of macrophages to PLA degradation products, metabolic reprogramming is characterized by concomitantly elevated oxidative phosphorylation and glycolysis, resulting in increased IL‐6, MCP‐1, TNF‐α, and IL‐1β protein expression, potent proinflammatory cytokines. Increased glycolysis, a fundamental proinflammatory metabolic phenotype, is likely mediated by HIF‐1α.^[^ ^35^ ^]^ Human fibroblasts in lactate‐enriched medium stabilize HIF‐1α resulting in increased glycolysis^[^ ^45^ ^]^ which underlies the activation of fibroblasts in several profibrotic pathologies.^[^ ^18^ ^]^ Similarly, increased oxidative phosphorylation is required for macrophages to function as antigen‐presenting cells as part of inflammation^[^ ^46^ ^]^ or its resolution.^[^ ^19^ ^]^

Inhibiting different steps in the glycolytic pathway produced similar effects, decreasing proinflammatory cytokine expression by modulating metabolic reprogramming and altering bioenergetics. Unlike bacterial LPS‐mediated glycolytic reprogramming that is uniquely dependent on IL‐1β,^[^ ^35^ ^]^ PLA degradation products additionally affect IL‐6, MCP‐1, and TNF‐α. Of note, modulating proinflammatory cytokine expression using aminooxyacetic acid stimulated IL‐10 protein expression, an anti‐inflammatory cytokine.^[^ ^33^ ^]^ Collectively, these findings are important for at least four reasons. First, it explains the “Oppenheimer phenomenon”, where long‐term PLA implantation results in neoplasia in some humans and up to 80% of rodents^[^ ^6^ ^]^ since IL‐6 directly links persistent inflammation from PLA to cellular transformation.^[^ ^47^ ^]^ Second, stimulating IL‐10 is critical to tissue repair by driving wound resolution and angiogenesis.^[^ ^48^ ^]^ In fact, IL‐10 is a key immunomodulatory cytokine secreted by mesenchymal stem cells,^[^ ^49^ ^]^ and is crucial in macrophage‐stem cell crosstalk^[^ ^50^ ^]^ for tissue engineering. Third, macrophages that have normal metabolism are unaffected by the small molecule inhibitors studied. In fact, cytotoxicity is selective for macrophages having altered metabolism, following exposure to PLA degradation products, making this technique particularly desirable. Fourth, it provides a basis to study lactate signaling in tumor initiation, with the potential to stop neoplastic initiation.

In cell culture medium used in our studies, serum and high bicarbonate salts buffered the pH of PLA degradation products, excluding pH as a confounder of observed metabolic cellular changes. Furthermore, using monomeric l‐lactic acid at various concentrations that simulated neutralized and acidic PLA degradation products, we similarly observed bioenergetic alterations, excluding pH as a confounder. Lastly, using aminooxyacetic acid to modulate some adverse responses to PLA degradation products suggests that acidity is not solely the driver of immune cellular activation to PLA.

Our findings using sterile implants present a perspective different than what is observed with bacterial endotoxin (LPS). Within 1 h of exposure to very low endotoxin concentrations, significant metabolic changes characterized by increased glycolysis and decreased oxidative phosphorylation occurs^[^ ^34^ ^]^; with PLA degradation products, we only observed changes after several days of exposure, with distinct metabolism. Importantly, LPS decreases ATP levels^[^ ^34^, ^51^ ^]^; in contrast, PLA degradation products (including monomeric l‐lactic acid) increase ATP levels as shown in this study. Lastly, unlike PLA degradation products, LPS‐mediated glycolytic reprogramming is reliant on IL‐1β signaling.^[^ ^35^ ^]^

Lactate is a signaling molecule in immunity^[^ ^52^ ^]^ and cancer progression.^[^ ^53^ ^]^ Its role when combined with LPS is conflicting, with reports of proinflammatory and anti‐inflammatory effects.^[^ ^54^, ^55^ ^]^ However, a stand‐alone ability of lactate to activate immune cells is novel, as prior inflammatory and cancer models did not simulate prolonged exposure times, a critical feature of cancer and immune microenvironments.

Amorphous PLA, which undergoes faster hydrolytic degradation than crystalline PLA results in quicker onset of metabolic reprogramming. Nonetheless, crystalline PLA does eventually result in metabolic remodeling and altered bioenergetics. Furthermore, our data implicate monocarboxylate transporters which mediate the bi‐directional flux of lactate across cell membranes.^[^ ^32^, ^54^ ^]^

Glucose is the first substrate in glycolysis, a crucial bioenergetic pathway. As such, radiolabeled glucose (FDG) uptake is often used to measure glycolytic dependence, in vivo, such as in some cancers or inflammatory disorders where enhanced glycolysis is pivotal to disease progression.^[^ ^56^ ^]^ We observed increased glycolytic dependence in the PLA inflammatory microenvironment using sterile amorphous PLA, which was abrogated by 2DG, one of the glycolytic inhibitors applied in our in vitro studies. Unsurprisingly, after surgical resection of colorectal and cervical tumors in human patients, chronic, sterile inflammation from PLA‐based adhesion barriers elevate FDG uptake, falsely mimicking cancer recurrence.^[^ ^57^ ^]^

Surprisingly, 2DG did not significantly reduce macrophage recruitment as measured by the expression of CD11b or F4/80 in the PLA microenvironment. However, 2DG reduced macrophage activation into a proinflammatory phenotype (CD86), likely by competing with radiolabeled glucose for binding to hexokinase,^[^ ^35^ ^]^ thereby inhibiting the first step in glycolysis. Since hydroxyapatite (HA) is often used to neutralize acidity from PLA degradation,^[^ ^10^ ^]^ we compared the effects of incorporating similar amounts (w/w) of HA to 2DG toward clinical translation of techniques targeting metabolism. Compared to 2DG, we observed increased pro‐regenerative macrophage expression (CD206) with HA, which is consistent with the bioactivity of ceramic biomaterials,^[^ ^42^ ^]^ opening the possibility of combinatorial strategies for regenerative applications. Corroborating CD206 results, our in vitro data showed that neither 2DG nor 3PO, as a glycolytic inhibitor for PLA‐based application, increases IL‐4 or IL‐10.

Compared to inhibiting glycolysis using 2DG, neutralizing acidity using HA increased macrophage recruitment and proinflammatory polarization, suggesting that metabolism and not acidity, is at the center of adverse immune responses to bulk PLA implants and PLA degradation products. Contrary to some studies, an explanation for the inability of HA to reduce inflammation in our study could be the amount used. Whereas the w/w concentration of HA present in our fabricated composites was 2% for direct comparison to 2DG, > 20% HA concentrations are more often used.^[^ ^58^ ^]^ However, it is noteworthy that 2% HA resulted in significantly increased CD206 expression, suggesting pharmacological efficacy, yet could not reduce CD86 expression. Furthermore, unlike in soft tissue regeneration, enhanced mechanical properties of implants having more concentration of or comprising of only HA is desirable for bone tissue engineering.^[^ ^58^ ^]^

Increased fibroblast activation, measured by α‐SMA expression, in the PLA microenvironment was reduced by inhibiting glycolysis using 2DG and not neutralizing acidity using HA. Compared to HA, 2DG reduced both α‐SMA and TGF‐β expression, suggesting that underlying metabolism regulates fibroblast activation in the PLA microenvironment. In agreement, metabolic reprogramming is known to play a key role in profibrotic disorders, activating fibroblasts.^[^ ^18^ ^]^

Most, if not all, publications on PLA's biomedical application include a statement indicating that PLA breakdown products are metabolized through the tricarboxylic acid cycle. However, not until this study has it been demonstrated that bioenergetic changes occur in response to PLA. This key observation will redirect the field of tissue engineering, by offering an opportunity to intervene in this response. It opens up the possibility to computationally identifying relevant small molecules that could be clinically deployed, and embedded in PLA implants, to mitigate adverse responses after carefully tuning drug release profiles. Furthermore, our study provides the basis to identify methods to personalize the delivery of metabolic inhibitors for precision medical applications. Moreover, the use of PLA composites with ceramics could be optimized by combining the benefits of metabolic reprogramming with bioactivity of ceramics for bone tissue engineering. Beyond its ability to inhibit the uptake of glycolytic substrates, related glutamine metabolic pathways, affected by aminooxyacetic acid could be explored for driving pro‐regenerative macrophage response.^[^ ^59^ ^]^

## 4 Outlook

Taken together, our findings suggest a model where PLA degradation products, including monomers of l‐lactic acid, mechanistically remodel metabolism in cells of the immune microenvironment. This mechanism is specific and leads to increased proinflammatory cytokine and marker expression which can be modulated while stimulating anti‐inflammatory cytokines. Nonetheless, there remains many unaddressed questions, including identifying what mediators (e.g., reactive oxygen species, reduced nicotinamide adenine dinucleotide, nitric oxide, etc.) and receptors (e.g., monocarboxylate transporters) drive downstream signaling in the biomaterial micronenvironment whose metabolic state is changed. Also, while it is likely that they play a key role, it is not fully understood how oligomeric degradation products interact with immune cells, and if they bind to immunoglobulins to elicit immune responses. Given the foundational role of metabolism in several physiological processes in the body, it is yet to be seen how the interaction of immune cells and (mesenchymal) stem cells is affected by immunometabolism for regenerative medicine. Building on our findings, future studies addressing these questions have the potential to significantly advance the field of tissue engineering.

## 5 Experimental Section

### Polylactide (PLA) Materials and Extraction

Highly crystalline PLA 3100HP and amorphous PLA 4060D (both from NatureWorks LLC) were used after their physicochemical and thermal properties were authenticated (Table S1, Supporting Information). PLA was sterilized by exposure to UV radiation for 30 min.^[^ ^24^ ^]^ Afterward, breakdown products (extracts)^[^ ^20^ ^]^ of PLA were obtained by suspending 4 g of PLA pellets in 25 mL of complete medium. Complete medium comprised of DMEM medium, 10% heat‐inactivated Fetal Bovine Serum, and 100 U mL^−1^ penicillin–streptomycin (all from ThermoFisher Scientific). PLA was extracted for 12 days in an orbital shaker at 250 rpm and 37 °C, after which extracts were decanted and the extract's pH measured. Either 100 or 150 µL of extract (specified in each figure legend) was used per well of a flat‐bottom 96‐well plate; each volume was made up to 200 µL, as the final volume, using a freshly made complete medium.

### Bioenergetic Assessment

Bioluminescence was measured using the IVIS Spectrum in vivo imaging system (PerkinElmer) after adding 150 µg mL^−1^ of d‐luciferin (PerkinElmer). Living Image (Version 4.5.2, PerkinElmer) was used for acquiring bioluminescence on the IVIS Spectrum. Standard ATP/ADP kits (Sigma–Aldrich) containing d‐luciferin, luciferase, and cell lysis buffer were used to according to manufacturer's instructions. Luminescence at integration time of 1000 ms was obtained using the SpectraMax M3 Spectrophotometer (Molecular Devices) using SoftMax Pro (Version 7.0.2, Molecular Devices).

### pH Measurements

The pH of extracts was assessed using an Orion Star A111 Benchtop pH Meter (ThermoFisher Scientific) under room temperature conditions (20 °C).

### Microscopy

Z‐stack microscopy was accomplished by using the DeltaVision deconvolution imaging system (GE Healthcare) and softWoRx software (Version 7.2.1, GE Healthcare) at excitation and emission wavelengths of 525 and 558 nm, respectively, for FITC. Section thickness of 0.2 µm for 64–128 sections were obtained at 40× and 100× magnification while imaging. Chambered Coverglass (Nunc Lab‐Tek II) was used to seed 20 000 BGL cells (see cells below), keeping similar ratios as in a 96‐well plate for volume of PLA extracts to volume of fresh medium.

### Glucose Measurement

Glucose levels in complete medium were evaluated by a hand‐held GM‐100 glucose meter (BioReactor Sciences) after validation (Figure S1b, Supporting Information) according to manufacturer's instruction.

### Cells

Mouse embryonic fibroblast cell line (NIH 3T3 cell line; ATCC) and murine primary bone‐marrow‐derived macrophages (BMDMs) were used. In each experiment, either 5000 fibroblasts or 50 000 BMDMs were initially seeded. BMDMs were sourced from male and female C57BL/6J mice (Jackson Laboratories) of 3–4 months.^[^ ^29^, ^34^ ^]^ NIH 3T3 cells were stably transfected with a Sleeping Beauty transposon plasmid (pLuBIG) having a bidirectional promoter driving an improved firefly luciferase gene (fLuc) and a fusion gene encoding a Blasticidin‐resistance marker (BsdR) linked to eGFP (BGL)^[^ ^25^ ^]^; enables to simultaneously monitor morphological and bioenergetic changes in live cells.^[^ ^60^ ^]^ All cells were cultured in a total of 200 µl complete medium with volumes of extracts specified in figure legends.

### Materials

3‐(3‐Pyridinyl)−1‐(4‐pyridinyl)−2‐propen‐1‐one (MilliporeSigma), 2‐deoxyglucose (MilliporeSigma) and aminooxyacetic acid (Sigma–Aldrich) were used for glycolytic inhibition and l‐lactic acid (Sigma–Aldrich) was used at various concentrations to reproduce the effects of PLA degradation products. Each of these materials were made in complete medium before adding to wells of a 96‐well plate.

### Cell Viability

Cell viability was assessed using the crystal violet staining assay,^[^ ^28^ ^]^ at room temperature, as an end‐point measure of total biomass generated over the course of the culture period. Briefly, out of 200 µL of medium per well, 150 µL was discarded. To each well, 150 µL of 99.9% methanol (MilliporeSigma) was added for 15 min to kill and fix the cells, then discarded. Afterward, 100 µL of 0.5% crystal violet (25% methanol) was added for 20 min, then the wells were emptied. Each well was washed twice with 200 µL of phosphate‐buffered saline for 2 min. Absorbance (optical density) was acquired at 570 nm using the SpectraMax M3 Spectrophotometer (Molecular Devices) and SoftMax Pro software (Version 7.0.2, Molecular Devices).

### Functional Metabolism

Basal measurements of oxygen consumption rate (OCR), extracellular acidification rate (ECAR), and lactate‐linked proton efflux rate (PER) were obtained in real‐time using the Seahorse XFe‐96 Extracellular Flux Analyzer (Agilent Technologies).^[^ ^33^, ^34^, ^35^ ^]^ Prior to running the assay, the cell culture medium was washed with and replaced by the Seahorse XF DMEM medium (pH 7.4) supplemented with 25 mm d‐glucose and 4 mm Glutamine. The Seahorse plates were equilibrated in a non‐CO_2_ incubator for 1 h prior to the assay. The Seahorse ATP rate and cell energy phenotype assays were run according to manufacturer's instruction and all reagents for the Seahorse assays were sourced from Agilent Technologies. Wave software (Version 2.6.1) was used to export Seahorse data directly as means ± standard deviation (SD).

### Chemokine and Cytokine Measurements

Cytokine and chemokine levels were measured using a MILLIPLEX MAP mouse magnetic bead multiplex kit (MilliporeSigma)^[^ ^38^ ^]^ to assess for IL‐6, MCP‐1, TNF‐α, IL‐1β, IL‐4, IL‐10, IFN‐γ, and 1L‐13 protein expression in supernatants. Data was acquired using Luminex 200 (Luminex Corporation) by the xPONENT software (Version 3.1, Luminex Corporation). Using the glycolytic inhibitor, 3PO, expectedly decreased cytokine values to < 3.2 pg mL^−1^ in some experiments. For statistical analyses, those values were expressed as 3.1 pg mL^−1^. Values exceeding the dynamic range of the assay, in accordance with manufacturer's instruction, were excluded. Additionally, IL‐6 ELISA kits (RayBiotech) for supernatants were used according to manufacturer's instructions.

### d/l‐Lactic Acid Determination Assays

Measurements of l‐ and d‐lactic acid were using standard d‐ and l‐lactate assay kits (Sigma–Aldrich) according to manufacturer's instruction after optimization (Figure S5a,b, Supporting Information). Negative absorbance values which were outside the dynamic range for the assay were excluded during analysis.

### Liquid Chromatography‐Electrospray Ionization Mass Spectrometry (LC‐ESI‐MS)

PLA extracts made in Milli‐Q water were analyzed by LC–ESI–MS by modifying a previously described method.^[^ ^61^ ^]^ Extracts were assessed using a Q‐Exactive mass spectrometer interfaced with a Thermo Vanquish UHPLC. One injected 5 uL of extract onto a Waters Acquity BEH‐C18 UPLC column (2.1 × 100 mm) and lactic acid oligomers were separated using the following gradient: initial conditions were 98% mobile phase A (0.1% formic acid in water) and 2% mobile phase B (acetonitrile + 0.1% formic acid), hold at 2% B until 1.0 min, linear ramp to 99% B at 7.0 min and hold at 99% B until 8 min, return to 2% B at 8.1 min and hold at 2% B until 10 min. While the flow rate was set at 0.3 mL min^−1^, the column temperature was 40 °C. Ions were generated by electrospray ionization in negative mode with a capillary voltage of −2.5 kV and source gas flow and temperature settings were set as the source auto‐defaults for an LC flow rate of 0.3 mL min^−1^. MS and MS/MS data were acquired using a data‐dependent MS method with survey scans acquired at 70 000 resolution (scan range m/z 80–1200) and MS/MS scans for the top five ions acquired at 17 500 resolution with an isolation width of 1.0 m/z and stepped normalized collision energies settings of 10, 30, and 60.

### Optical Rotation

Polarimetry was used to characterize the L‐content and optical purity of the PLA samples with a P‐2000 polarimeter (Jasco) by the Spectra Manager software (Version 2.13.00, Jasco). The optical rotation, [𝛼]_25_, was measured and averaged for three samples of each polymer in chloroform (Omnisolv), at a concentration of 1 g mL^−1^. Conditions were set at 25 °C and 589 nm wavelength. Sucrose was used as a standard reference material, and its specific optical rotation was reported as ≈ 67°.

### Gel Permeation Chromatography

Gel permeation chromatography (GPC) was conducted to characterize the polymer molecular weights using a 600 controller (Waters) equipped with Optilab T‐rEX refractive index (RI) and TREOS II multi‐angle light scattering (MALS) detectors (Wyatt Technology Corporation), and a PLgel 5 µm MIXED‐C column (Agilent Technologies) with chloroform eluent (1 mL min^−1^). ASTRA software (Version 7.3.2.21, Wyatt Technology Corporation) was used. Polystyrene standards (Alfa Aesar) with M_n_ ranging from 35 000 to 900 000 Da were used for calibration.

### Differential Scanning Calorimetry

Differential scanning calorimetry (DSC) was conducted with a DSC Q20 (TA Instruments) to analyze the melting temperature (*T*_m_), glass transition temperature (T_g_), and percent crystallinity of the PLA grades. Thermal Advantage software (Version 5.5.23, TA Instruments) was used. The temperature was first equilibrated to 0 °C, then ramped up to 200 °C at a heating rate of 10 °C min^−1^; the temperature was then held isothermally for 5 min. Afterward, the sample was cooled back to 0 °C at a rate of 10 °C min^−1^, then held isothermally for 2 min. Finally, the material was heated back to 200 °C at 10 °C min^−1^.

### In Vivo Studies, Tissue Processing, and Analyses

Amorphous PLA was compounded with 2DG at 190 °C for 3 min in a DSM 15 cc mini‐extruder (DSM Xplore) and pelletizer (Leistritz Extrusion Technology). The in‐vitro studies indicate 1 mm 2DG to be an effective concentration. Accordingly, one estimated that 189 mg of 2DG in 10 g of amorphous PLA will approximate effective concentrations after accounting for the potential thermal degradation of 2DG, converting mm to w/w values.^[^ ^62^ ^]^ Comparable amounts (200 mg) of hydroxyapatite (HA; 2.5 µm particle sizes^[^ ^40^ ^]^; Sigma–Aldrich) in were compounded 10 g of amorphous PLA under the same melt‐blending thermal conditions. To exclude the effect of melt‐blending as a confounder in studies, amorphous PLA controls were processed under the same thermal conditions to make “reprocessed” amorphous PLA. Pellets from melt‐blending were made into 1.75 mm diameter filaments using an extruder (Filabot EX2) at 170 °C with air set at 93. For surgical implantation, amorphous PLA filaments were cut into 1 mm lengths; four biomaterials were subcutaneously implanted on the dorsum (back) of each mouse, with two cranially (2.5 cm apart) and two caudally (2.5 cm apart).^[^ ^12^ ^]^

Two‐month‐old female C57BL/6J mice (n = 3 mice per group) with an average weight of 19 g were used according to procedures approved by the Institutional Animal Care and Use Committee at Michigan State University (PROTO202100327). Mice were anesthetized using isoflurane (2%–3%). The back of each mouse was shaved and alternate iodine and alcohol swabs were used as skin disinfectants. Aseptic surgery consisted of incisions through the skin into the subcutis, where biomaterials were inserted into a pouch made with forceps. Afterward, surgical glue (3 m Vetbond) was used to appose the skin. Each mouse received intraperitoneal or subcutaneous pre‐ and post‐operative meloxicam (5 mg kg^−1^) injections as well as postoperative saline. Sham controls underwent the same procedure without biomaterial implantation. After 6 weeks, the dorsum of mice was shaved to visibly observe sites of surgical implantation. Thereafter, mice were intraperitoneally injected with 4.82 MBq F‐18 fluorodeoxyglucose (Cardinal Health) in 200 µL. At 65 min post‐dose, mice were euthanized and blood drawn from their hearts. Circular biopsies (12 mm diameter) of full skin thickness, with visible implants in the center, were recovered. Similar‐sized biopsies were collected from mice in the sham group in the region where surgical incision was made. Biomaterial migration from subcutaneous sites only allowed for the recovery of most and not all implants. As such, for obtaining data on the gamma counter (Figure 7a), there were 12 skin biopsies from three mice in the sham group, 8 skin biopsies from three mice (amorphous PLA group), and 10 skin biopsies from three mice (amorphous + 2DG group). Skin biopsies, blood sample and heart organs were weighed, with only skin samples fixed in 4% paraformaldehyde (PFA). Activity in all samples was assessed via gamma counter (Wizard 2, Perkin Elmer) once decayed to a linear range. All injected doses and gamma counter measurements were decay‐corrected to the same timepoint to calculate the percent of injected dose taken up per gram of assessed tissue (%ID g^−1^; Figure 7a).

For tissue staining, one skin biopsy per mouse was passed through increasing concentration of 10%, 20%, and 30% sucrose, daily. Using 99.9% methanol (Sigma–Aldrich) on dry ice, tissues were embedded in optimal cutting temperature (O.C.T.) compound (Tissue‐Tek) by snap freezing. After equilibration at −20 °C, multiple successive 8 µm sections were obtained using a microtome‐cryostat. Sections were routinely stained using hematoxylin and eosin. Two different tissue sections were immunostained using conjugated antibodies as follows: 1) F4/80‐FITC (1:100; BioLegend; 123 107), CD11b‐PE (1:100; BioLegend; 101 207), CD206‐BV421 (1:200; BioLegend; 141 717) and CD86‐Alexa Fluor 647 (1:100; BioLegend; 105 019) using ordinary mounting medium; 2) alpha‐SMA‐eFluor660 (1:150; ThermoFisher Scientific; 50‐9760‐82), TGF‐beta‐PE (1:100; ThermoFisher Scientific; 12‐9821‐82) using DAPI mounting medium. Sections for TGF‐beta were permeabilized using 0.1% Triton X in 1× PBS (PBST) for 8 min then washed off with 1x PBS generously. Afterward, blocking buffer (0.5% bovine serum albumin in 1× PBS) was used to cover slides for 30 min. Slides were then incubated in antibodies at 4 °C overnight. Subsequently, slides with tissue sections were washed in 1× PBS, and mounting medium applied.

Immunostained sections on slides were imaged using a Leica DMi8 Thunder microscope fitted with a DFC9000 GTC sCMOS camera and LAS‐X software (Leica, version 3.7.4). Imaging settings at 20× magnification and 100% intensity were: 1) F4/80‐FITC excitation using the 475 laser (filter 535/70; 500 ms); CD11b‐PE excitation using the 555 laser (no filter; 500 ms); CD206‐BV421 excitation using 395 laser (no filter; 150 ms); CD86‐Alexa Fluor 647 excitation using the 635 laser (no filter; 500 ms). 2) alpha‐SMA‐eFluor660 excitation using the 635 laser (no filter; 500 ms), TGF‐beta‐PE excitation using the 555 laser (no filter; 500 ms) and DAPI excitation using the 395 laser (535 filter; 500 ms). On the other hand, sections stained with hematoxylin and eosin were imaged at 40× using the Nikon Eclipse Ci microscope fitted with a CoolSNAP DYNO (Photometrics) and NIS elements BR 5.21.02 software (Nikon Instruments Inc.). Microscope images were prepared and analyzed using ImageJ (version 1.53k). For analyzing immunostained sections, five randomly selected rectangular areas of interest (1644.708 µm^2^), encompassing cells adjacent to implants, were obtained as mean gray values^[^ ^63^ ^]^ a tissue section. In the sham group, biopsies were taken from incision sites, and areas without cells were also analyzed. Where derived from n = 2 or n = 3 mice, 10 or 15 data points, respectively were graphically represented to fully reveal inherent variance across samples (Figures 7b–e and 8a,b); only the aPLA + HA group had sections derived from n = 2 mice after one sample was damaged during cryo‐sectioning and excluded from analyses. Representative images (16‐bit; 0 to 65535) were adjusted to enhance contrast for direct comparison using ImageJ as follows: CD86 (800–11000), CD206 (2000–5000), F4/80 (500–4000), CD11b (800–11000), α‐SMA (1300–5000), DAPI (6000–31, 000), and TGF‐β (1900–13000).

Statistical software (GraphPad Prism) was used to analyze data presented as mean with standard deviation (SD). The significance level was set at *p* < 0.05, and details of statistical tests and sample sizes, which were biological replicates, are provided in figure legends. Exported data (mean, SD) from Wave in Seahorse experiments had the underlying assumption of normality and similar variance and thus were tested using corresponding parametric tests as indicated in figure legends.

## Conflict of Interest

C.V.M. and C.H.C. are inventors on a pending patent application filed by Michigan State University on metabolic reprogramming to biodegradable polymers.

## Author Contributions

Conceptualization, C.V.M. and C.H.C.; Methodology, C.V.M., K.R.Z., K.D.H., S.B.G., R.N. and C.H.C.; Investigation, C.V.M., M.A., E.U., M.O.B., M.M.K., K.S., A.V.M., H.P., S.C., J.M.H, C.L.M., S.J.C., M.H. and A.T.; Writing—Original Draft, C.V.M.; Writing—Review & Editing, C.V.M., M.A., E.U., M.O.B., M.M.K., K.S., A.V.M., H.P., S.C., J.M.H, C.L.M., S.J.C., M.H., A.T., K.R.Z., K.D.H., S.B.G., R.N. and C.H.C.; Funding Acquisition, C.H.C.; Resources, R.N. and C.H.C.; Supervision, K.R.Z., K.D.H., S.B.G., R.N. and C.H.C.

## Supporting information

Supporting Information

## Data Availability

The data that support the findings of this study are available from the corresponding author upon reasonable request.
