# Supplementary material for: Polylactide Degradation Activates Immune Cells by Metabolic Reprogramming
Source: Adv Sci (Weinh). 2023 Sep 22;10(31):2304632. doi: 10.1002/advs.202304632 (PMC10625072; doi:10.1002/advs.202304632)
Supplement: Supplementary file 1 — Supporting Information [file ADVS-10-2304632-s001.pdf]

ADVANCED SCIENCE
Open Access

Supporting Information

for *Adv. Sci.*, DOI 10.1002/advs.202304632

Polylactide Degradation Activates Immune Cells by Metabolic Reprogramming

*Chima V. Maduka, Mohammed Alhaj, Evran Ural, Oluwatosin M. Habeeb, Maxwell M. Kuhnert, Kylie Smith, Ashley V. Makela, Hunter Pope, Shoue Chen, Jeremy M. Hix, Christiane L. Mallett, Seock-Jin Chung, Maxwell Hakun, Anthony Tundo, Kurt R. Zinn, Kurt D. Hankenson, Stuart B. Goodman, Ramani Narayan and Christopher H. Contag**

ADVANCED SCIENCE
Open Access

Supporting Information

for *Adv. Sci.*, DOI 10.1002/advs.202304632

Polylactide Degradation Activates Immune Cells by Metabolic Reprogramming

*Chima V. Maduka, Mohammed Alhaj, Evran Ural, Oluwatosin M. Habeeb, Maxwell M. Kuhnert, Kylie Smith, Ashley V. Makela, Hunter Pope, Shoue Chen, Jeremy M. Hix, Christiane L. Mallett, Seock-Jin Chung, Maxwell Hakun, Anthony Tundo, Kurt R. Zinn, Kurt D. Hankenson, Stuart B. Goodman, Ramani Narayan and Christopher H. Contag**

Supporting Information

Polylactide Degradation Activates Immune Cells by Metabolic Reprogramming

*Chima V. Maduka, Mohammed Alhaj, Evran Ural, Oluwatosin M. Habeeb, Maxwell M. Kuhnert, Kylie Smith, Ashley V. Makela, Hunter Pope, Shoue Chen, Jeremy M. Hix, Christiane L. Mallett, Seock-Jin Chung, Maxwell Hakun, Anthony Tundo, Kurt R. Zinn, Kurt D. Hankenson, Stuart B. Goodman, Ramani Narayan, Christopher H. Contag**

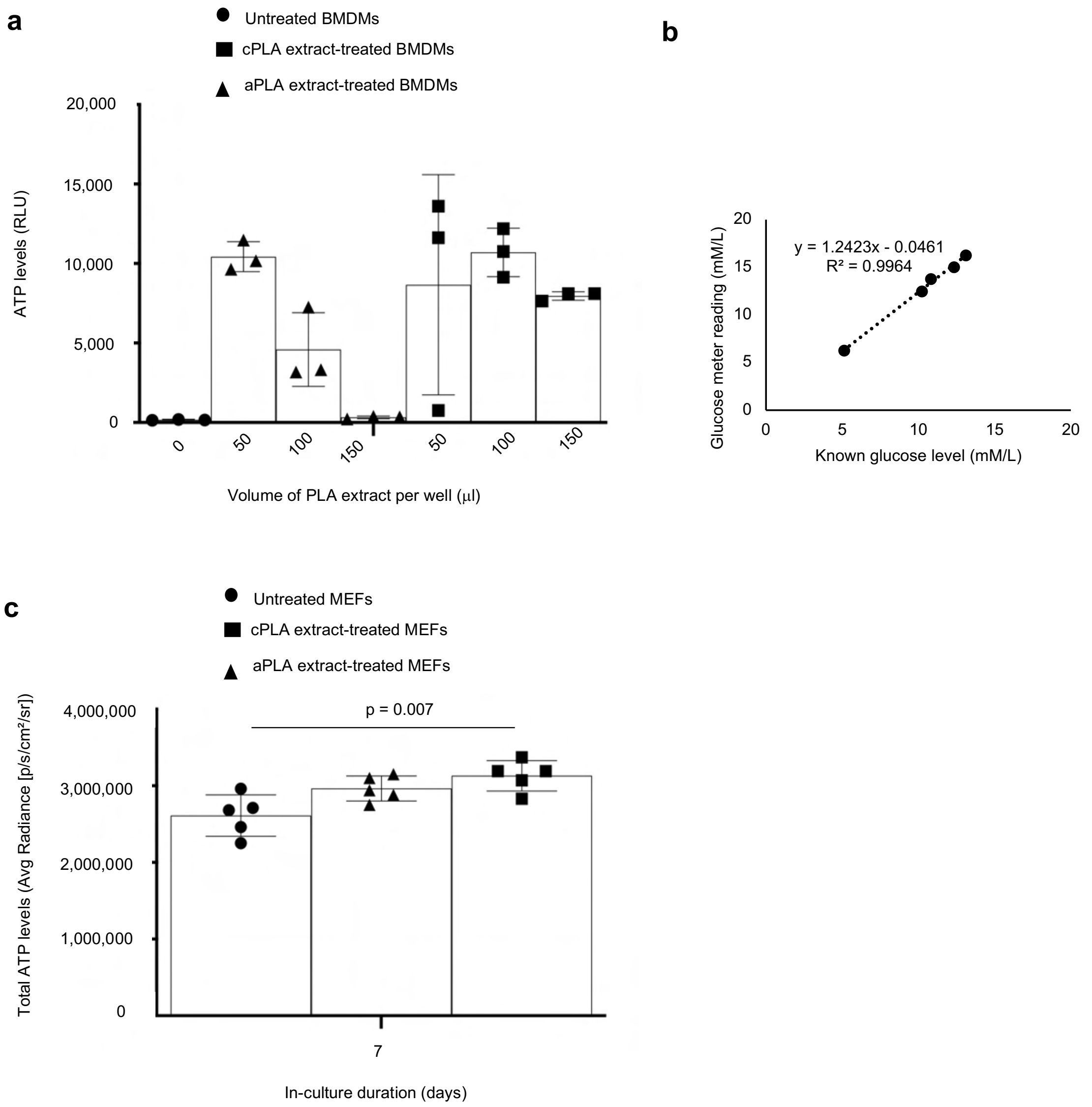

Figure S1. Different doses of polylactide (PLA) extract alter bioenergetic (ATP) levels in primary bone marrow-derived macrophages (BMDMs) and using the glucose meter can measure glucose levels in cell culture medium. a, Dose-bioenergetic response of the different PLA extracts on BMDMs revealed tendencies to alter ATP levels for all tested doses. b, Known glucose levels in cell culture medium linearly correlated (R square = 0.9964) with measurements from the glucose meter. c, Bioenergetic (ATP) levels are higher in mouse embryonic fibroblasts (MEFs) exposed to PLA extracts in comparison to controls. Mean (SD), n = 3 (Supplementary Fig. 1a), n = 5 (Supplementary Fig. 1c), simple linear regression, one-way ANOVA followed by Tukey's post-hoc test; crystalline PLA (cPLA), amorphous PLA (aPLA); 100 µl of control or PLA extract was used in Supplementary Fig. 1c.

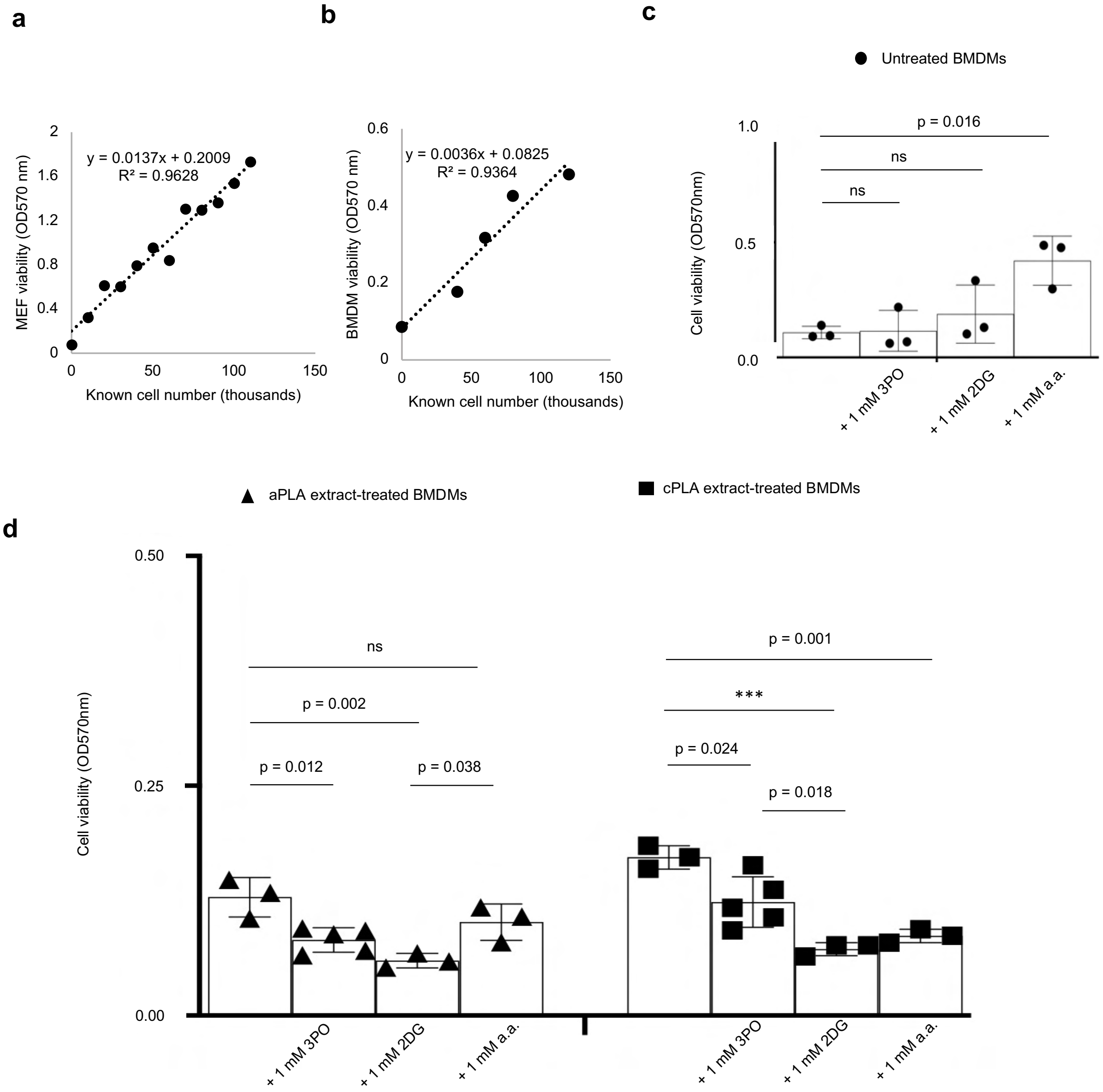

Figure S2. Crystal violet assay can measure cell viability and cytotoxicity was selective for cells exposed to polylactide (PLA) following treatment with glycolytic inhibitors. a-b, Known cell numbers linearly correlated with absorbance for a, mouse embryonic fibroblasts (MEF; R square = 0.9628) and b, primary bone marrow-derived macrophages (BMDMs; R square = 0.9364). c-d, Although cell viability was not decreased in untreated BMDMs following exposure to glycolytic inhibitors (c), BMDMs exposed to amorphous PLA (aPLA) or crystalline PLA (cPLA) degradation products (extract) decreased in cell viability after treatment with glycolytic inhibitors (d). Not significant (ns), *** $p < 0.001$, mean (SD), n = 3 (Supplementary Fig. 2c), n = 3-5 (Supplementary Fig. 2d), one-way ANOVA followed by Tukey's post-hoc test; 3-(3-pyridinyl)-1-(4-pyridinyl)-2-propen-1-one (3PO), 2-deoxyglucose (2DG) and aminooxyacetic acid (a.a.); 100 µl of control or PLA extract was used on day 7.

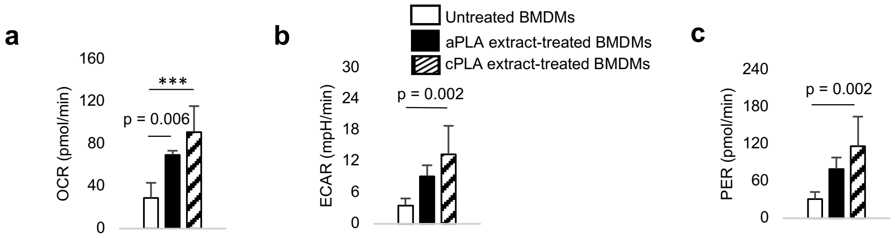

Figure S3. Functional metabolic indices are increased in primary bone marrow-derived macrophages (BMDMs) after exposure to crystalline PLA (cPLA) degradation products (extracts). a-c, Oxygen consumption rate (OCR, a), extracellular acidification rate (ECAR, b) and proton efflux rate (PER, c) are increased following exposure to cPLA extracts. *** $p < 0.001$, mean (SD), n=5, one-way ANOVA followed by Tukey's post-hoc test; 150 µl of control or PLA extract was used on day 7.

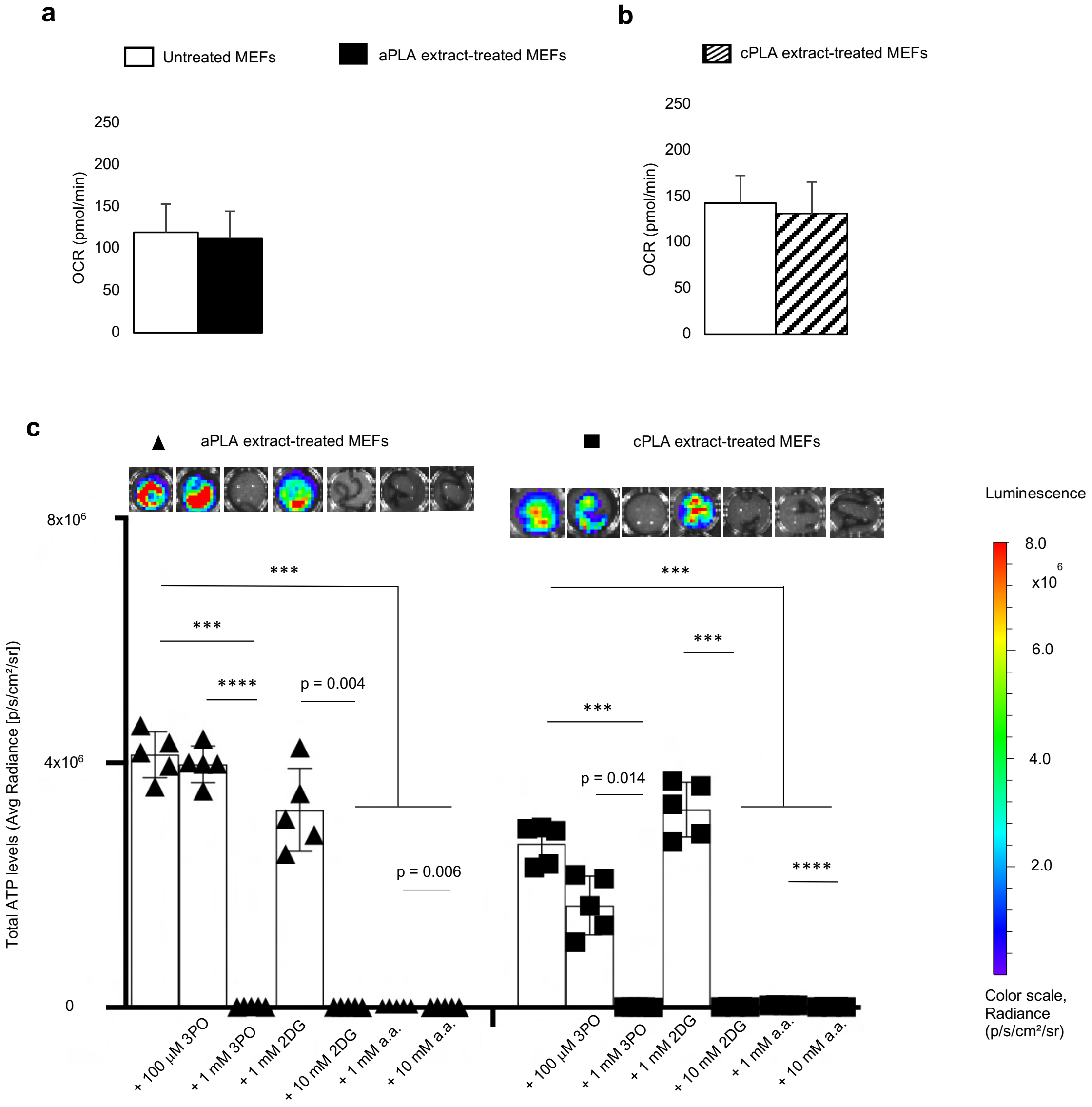

Figure S4. Oxygen consumption rate (OCR) is not altered in mouse embryonic fibroblasts (MEFs) following prolonged exposure to polylactide (PLA) degradation products (extract). a-b, Following exposure to amorphous PLA (aPLA; a) or crystalline PLA (cPLA; b) extracts, OCR is unaffected. c, Bioenergetic levels on day 12 in MEFs exposed to aPLA or cPLA extracts are decreased in a dose-dependent manner by 3-(3-pyridinyl)-1-(4-pyridinyl)-2-propen-1-one (3PO), 2-deoxyglucose (2DG) and aminooxyacetic acid (a.a.). *** $p < 0.001$, **** $p < 0.0001$, mean (SD), n = 3 (Supplementary Fig. 4a, b), n = 5 (Supplementary Fig. 4c), two-tailed unpaired t-test or Brown-Forsythe and Welch ANOVA followed by Dunnett's T3 multiple comparisons test; 100 µl of control or PLA extract was used on day 7 (Supplementary Fig. 4a, b) or 12 (Supplementary Fig. 4c).

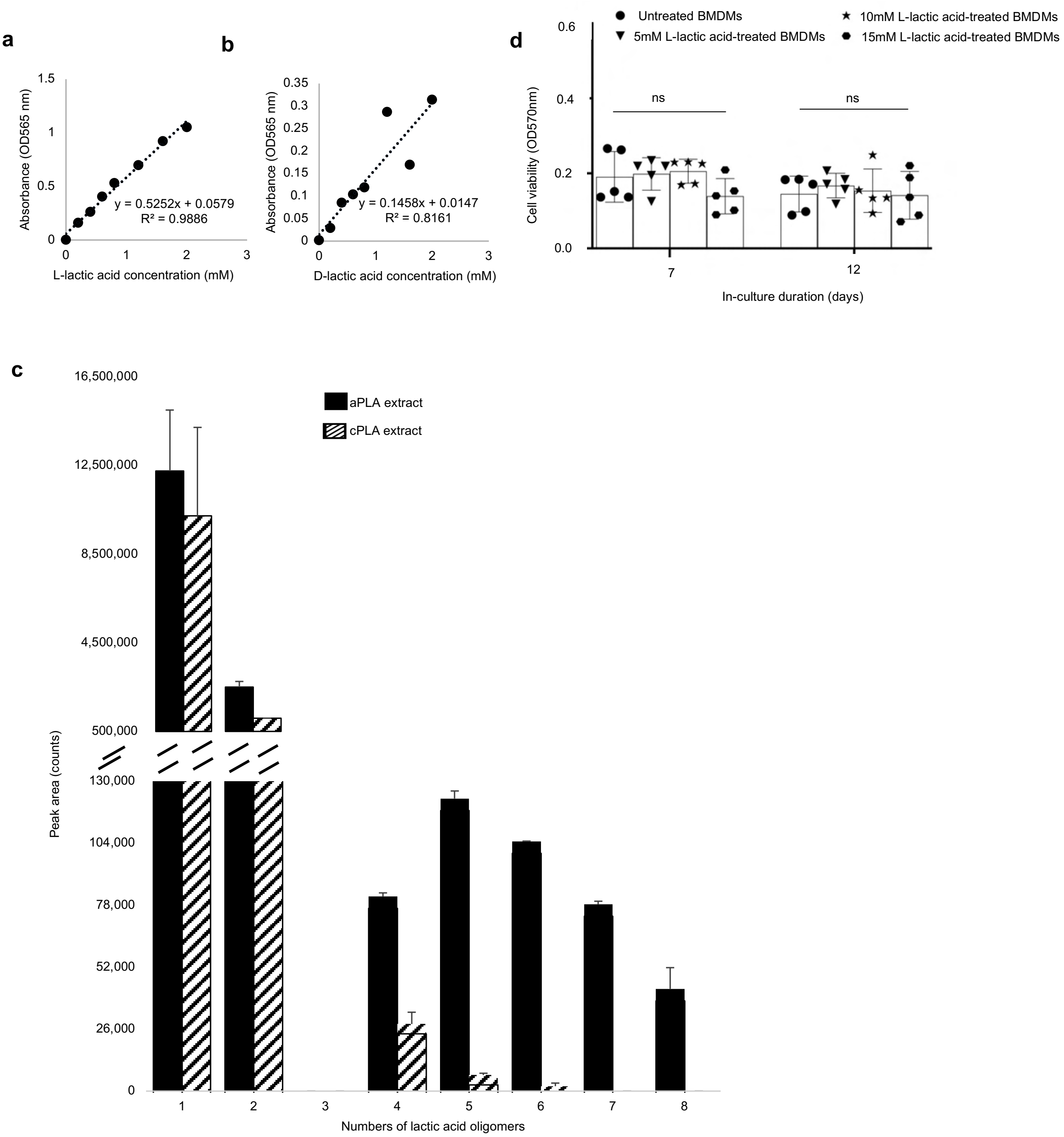

Figure S5. D- and L-lactic acid levels can be detected by absorbance and cell viability is similar among macrophages treated with L-lactic acid. a-b, Known concentrations of L-lactic (R square = 0.9886; a) and D-lactic (R square = 0.8161; b) acid linearly correlate with absorbance. c, Liquid chromatography-electrospray ionization mass spectrometry amorphous polylactide (aPLA) and crystalline polylactide (cPLA) extracts derived in milliQ-water. d, Viability of primary bone marrow-derived macrophages (BMDMs) is similar after treatment with L-lactic acid over time. Not significant (ns), one-way ANOVA, mean (SD), n=2 (Supplementary Fig. 5c), n=5 (Supplementary Fig. 5d).

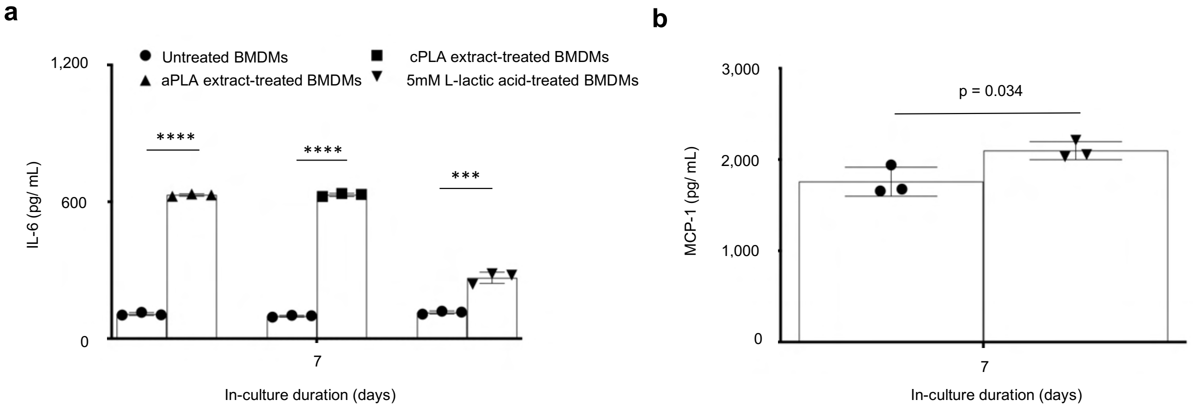

Figure S6. IL-6 and MCP-1 protein levels are increased following prolonged exposure of primary bone marrow-derived macrophages (BMDMs) to L-lactic acid in comparison to untreated BMDMs. a, Using ELISA reproduced changes in IL-6 levels following exposure of BMDMs to amorphous PLA (aPLA), crystalline PLA (cPLA) or L-lactic acid. b, Similarly, MCP-1 levels are increased after exposing BMDMs to L-lactic acid as measured by the MILLIPLEX assay. ***p<0.001, ****p<0.0001, mean (SD), n=3, two-tailed unpaired t-test; 100 µl of aPLA or 150 µl of cPLA with corresponding controls were used; whereas corresponding controls for PLA were incubated for 12 days, the controls for L-lactic acid were not.

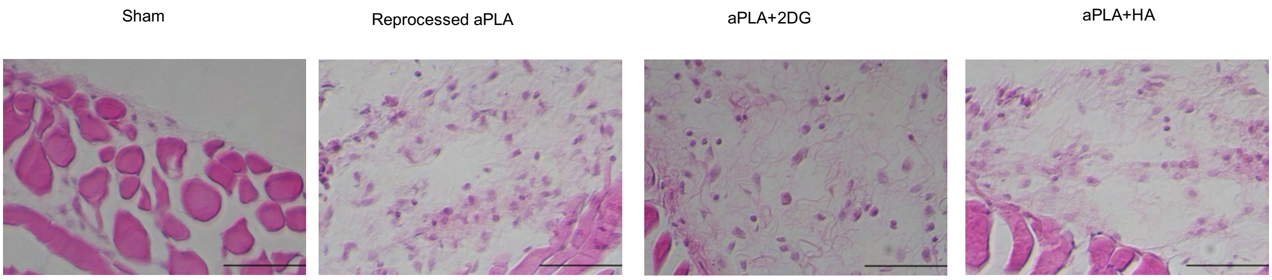

Figure S7. Recruitment of inflammatory cellular infiltrates following implantation of amorphous polylactide (aPLA) with and without 2-deoxyglucose (2DG) or hydroxyapatite (HA) is compared to sham controls in cryo-sections stained using hematoxylin/ eosin (scale bars, 20 μm).

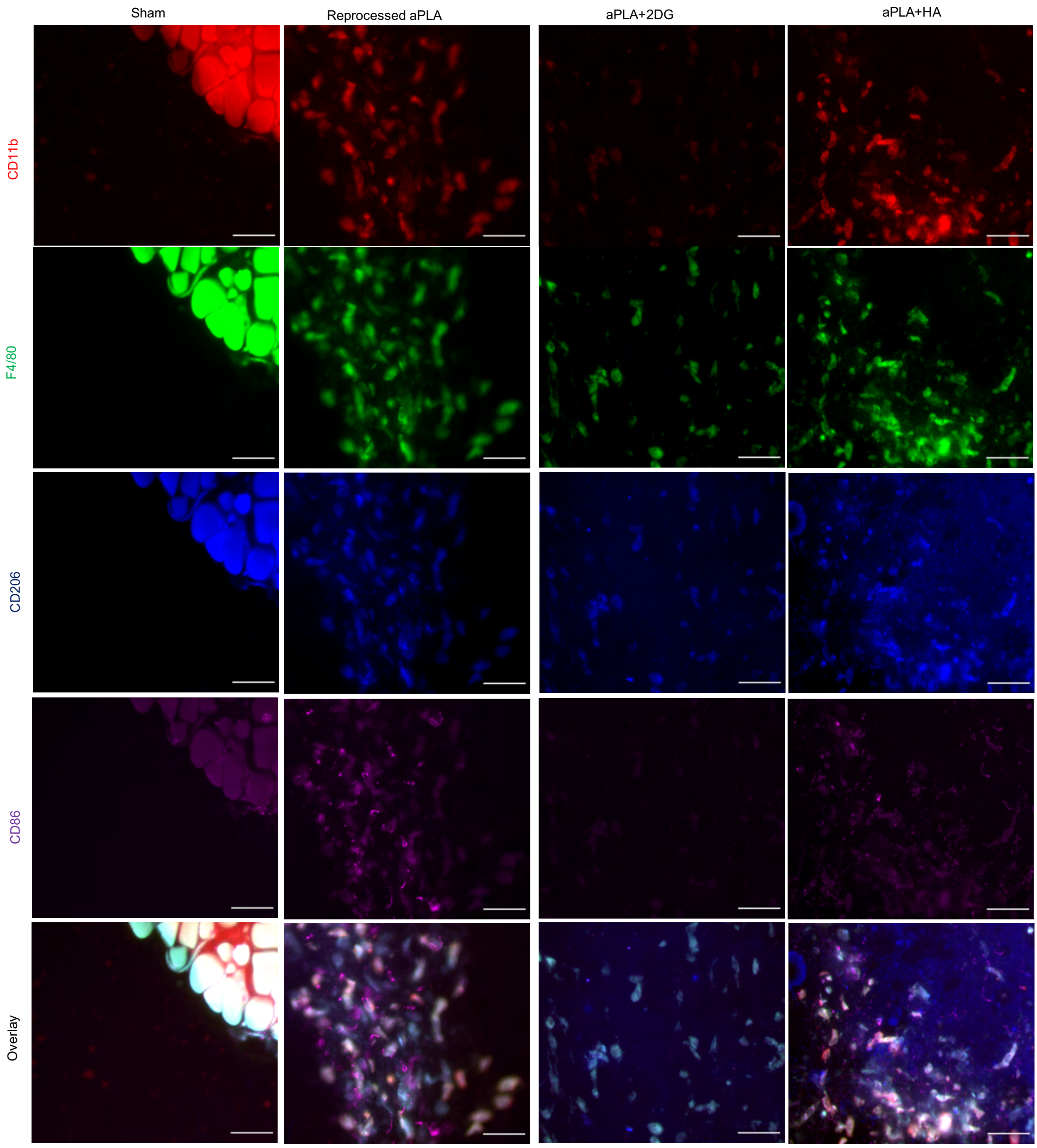

Figure S8. Immunohistochemical staining with CD11b-PE, F4/80-FITC, CD206-BV421 and CD86-AF647 reveal the presence and polarization of macrophages following implantation of amorphous polylactide (aPLA) with and without 2-deoxyglucose (2DG) or hydroxyapatite (HA) when compared to sham controls (scale bars, 50 μm).

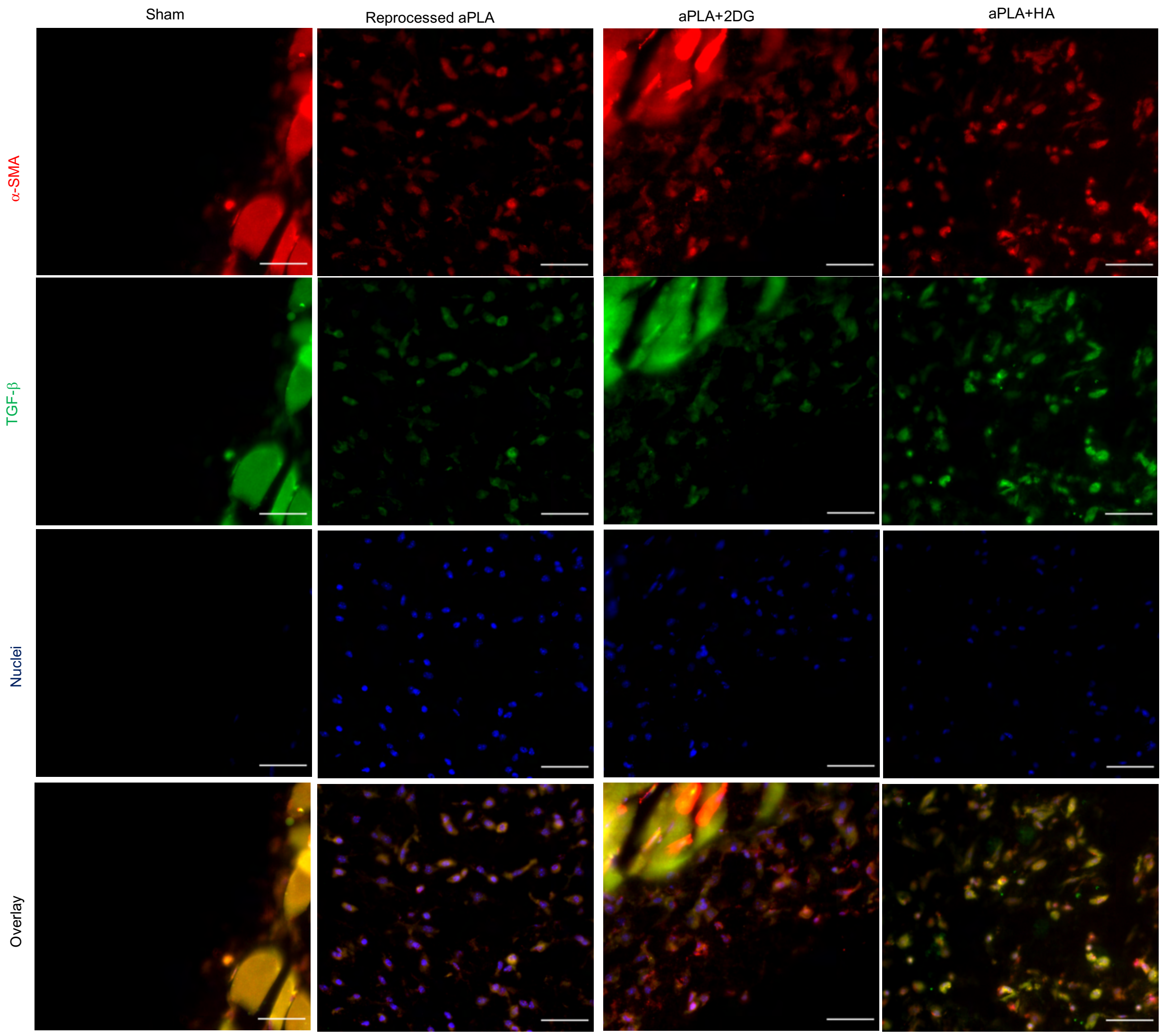

Figure S9. Immunohistochemical staining with α-SMA-eFluor 660 and TGF-β-PE using a DAPI mounting medium show fibroblast activation following implantation of amorphous polylactide (aPLA) with and without 2-deoxyglucose (2DG) or hydroxyapatite (HA) when compared to sham controls (scale bars, 50 μm).

Table S1. Authentication of physicochemical and thermal properties of commercial polylactide (PLA).

Criteria	PLA 3100HP (Crystalline PLA)	PLA 4060D (Amorphous PLA)
Optical purity (%)	99.04	81.47
L-content (%)	99.40	90.54
Glass transition temperature T_g (°C)[‡]	62.20	59.05
Melting temperature T_m (°C)[‡]	175.85	N/A
Crystallinity (pellet, %)[†]	51.46	0
Crystallinity (resin, %)[‡]	42.49	0
Number average molecular weights M_n (Da)	87,390	113,270
Weight average molecular weights M_w (Da)	157,060	200,200
Polydispersity index	1.797	1.767

[†]Percentage crystallinity of pellets was determined based on the first heating cycle.
[‡]Percentage crystallinity of the resin, T_g, and T_m were determined based on the second heating cycle.
Molecular weights were based on a calibration curve of polystyrene standards.

Table S2. Molecular weights of polylactide (PLA) samples decrease after extraction in medium or water.

Extracted in:		Initial molecular weight (Da)		Final molecular weight (Da)		Decrease (%)	
	PLA sample	M_n	M_w	M_n	M_w	M_n	M_w
Serum-containing medium	cPLA	87,390 ± 2,840	157,060 ± 3,640	75,155 ± 1,340	140,540 ± 2,390	14.0	10.5
	aPLA	113,270 ± 1,880	200,200 ± 2,150	100,923 ± 3,380	185,365 ± 3,900	10.9	7.4
Milli-Q water	cPLA	--	--	75,155 ± 1,340	140,540 ± 2,390	14.0	10.5
	aPLA	--	--	103,302 ± 2,180	185,250 ± 3,560	8.8	7.5

Number average molecular weight (M_n) and weight average molecular weight (M_w) are expressed as mean (SD) and based on a calibration curve of polystyrene standards, n=3; crystalline PLA (cPLA), amorphous PLA (aPLA); Dashed line indicates that initial weights are the same for PLA, irrespective of whether PLA will be extracted in water or complete medium.

Table S3. Monomers of L- and D-lactic acid are detectable in extracts of polylactide.

	L-lactic acid (OD565nm)	D-lactic acid (OD565nm)
Crystalline PLA (cPLA) extract	0.0034 ± 0.0025	0.0021 ± 0.0010
Amorphous PLA (aPLA) extract	0.0051 ± 0.0036	0.0023 ± 0.0002
Control (milli-Q water)	0.0007 ± 0.0004	0.0008 ± 0.0001

Lactic acid absorbance is expressed as mean (SD), n=2-3.
